# Natural infection of hybrid sturgeon (*Acipenser baerii*♀×*Acipenser schrenckii*♂) with *Nocardia seriolae* and white sturgeon iridovirus: pathological and transcriptomic analyses

**DOI:** 10.3389/fimmu.2024.1488159

**Published:** 2024-11-22

**Authors:** Luyun Ni, Pengcheng Li, Qiaolin Zou, Feiyang Li, Yeyu Chen, Haoting Chen, Jiansheng Lai, Jun Du, Ya Liu

**Affiliations:** ^1^ Fisheries Research Institute, Sichuan Academy of Agricultural Sciences, Chengdu, Sichuan, China; ^2^ Guangzhou Double Helix Gene Technology Co., Ltd., Guangzhou, Guangdong, China

**Keywords:** sturgeon, Nocardia, iridovirus, transcriptome, immune response

## Abstract

**Introduction:**

In August 2023, hybrid sturgeons (*Acipenser baerii♀×Acipenser schrenckii♂*) cultured in Sichuan, China, showed infectious disease symptoms, including ulcers, liver and spleen nodules, and high mortality rates.

**Methods:**

Pathogenic bacteria were isolated from the liver of diseased sturgeons and analyzed for their phenotypic and molecular traits. Furthermore, iridovirus-specific TaqMan real-time PCR (RT-PCR) analyses were conducted. The histopathological characteristics were analyzed using paraffin sectioning and transmission electron microscopy (TEM). Transcriptome sequencing was performed to elucidate the impact of pathogen exposure and immune response profiles in infected sturgeon.

**Results:**

Pathogenic bacteria isolation and phylogenetic analyses of the *16S rRNA* gene confirmed that the isolated bacteria clustered within the *Nocardia seriolae* group. The TaqMan RT-PCR assay was performed to detect the presence of white sturgeon iridovirus (WSIV), indicating a weakly positive signal. Histopathological examination revealed severe damage to various tissues, and a notable presence of bacteria was observed through acid-fast staining. Transmission electron microscopy analysis showed the presence of abundant bacteria and virus particles, indicating cellular invasion and subsequent damage. In summary, the disease in hybrid sturgeons was diagnosed as infection of *N. seriolae* and WSIV. To investigate the immune response of hybrid sturgeons to this infection, spleen transcriptomes were analyzed. Numerous immune-related genes and pathways, including the “Toll-like receptor”, “B-cell receptor”, and “T-cell receptor” signaling pathways, were altered in response to pathogenic threats. Significantly downregulated of key components of TCR and BCR signaling pathways, such as *ZAP70*, *BTK*, and *CD79A*, suggested a temporary inhibition of these pathways critical for cellular immunity post-infection. Gene set enrichment analysis revealed significant suppression of the apoptosis signaling pathway and activation of autophagy and mitophagy signaling pathways following infection. Specifically, in the death receptor-mediated apoptosis signaling pathway, downregulation of *TNFα*, *TRAIL*, *CASP6*, and *CASP8* was observed, while several genes in the autophagy and mitophagy pathways showed upregulated expression post-infection.

**Discussion:**

We report the initial occurrence of *N. seriolae* infection in cultured sturgeons. These findings could provide a theoretical basis for diagnosing and preventing this disease, as well as enhance the understanding of host-pathogen interactions in fish.

## 1 Introduction

Sturgeons, belonging to the order Acipenseriformes (Osteichthyes: Actinopterygii), are considered among the oldest and most primitive bony fishes still in existence (1). Unfortunately, 25 out of the 27 sturgeon species worldwide are currently facing extinction due to human activities (2). To address this, many countries have begun artificial breeding programs for sturgeons to both protect endangered species and meet market demands. China is currently leading in sturgeon breeding, with a focus on cultivating more than 13 species, including *Acipenser baerii*, *Acipenser schrenckii*, and various hybrid sturgeons, which collectively account for 90% of the total production and are the primary source of caviar (3). Rearing sturgeon in high-density facilities has increased both meat and caviar output but has also posed challenges with aquaculture techniques. A key obstacle to the growth of sturgeon aquaculture is the prevalence of contagious illnesses (4, 5). Bacterial diseases such as *Aeromonas veronii* (5), *Edwardsiella tarda* (6), and *Streptococcus iniae* (7), as well as viral diseases such as Missouri River sturgeon iridovirus (MRSIV), white sturgeon iridovirus (WSIV) and acipenserid herpesvirus-1 and 2 (AciHV-1 and AciHV-2) (8, 9), currently present significant challenges to global sturgeon farming. From an economic perspective, WSIV and AciHV-2 hold significant importance because of the potential financial losses they may cause. These diseases not only hinder conservation efforts for endangered sturgeon species but also threaten the sustainable development of the sturgeon breeding industry. In China, bacterial diseases are the primary concern in sturgeon farming, with viral diseases being less commonly reported.

Nocardiosis, caused by *Nocardia seriolae*, is a chronic granulomatous disease prevalent in humans and widely distributed globally. In aquaculture, *N. seriolae* can infect a diverse array of hosts, including yellowtails (10), largemouth bass (11), snakehead (12), snubnose pompano (13), grouper (14), as well as other freshwater and seawater fish. *N. seriolae* is recognized as an opportunistic pathogen. This opportunistic pathogen induces chronic and hidden infections in fish species. The main routes of infection include the gills, anus, lateral line, or through surface injuries, leading to symptoms characterized by numerous nodules on internal organs (15). Furthermore, infections caused by *N. seriolae* rarely lead to acute and widespread fish mortality; typically, the morbidity ranges from 15% to 30%, while cumulative mortality has been observed to reach up to 100% in certain instances. *N. seriolae* is categorized as a facultative intracellular, gram-positive pathogen displaying branching filamentous rods. In response to the invasion and dissemination of *N. seriolae*, the host organizes immune cells and epithelial cells around the affected areas, resulting in the formation of granulomatous tissues (16). Despite the significant impact of *N. seriolae* on fish farming, its pathogenic mechanisms remain inadequately understood. As for WSIV, it is a virus that infects the skin, gills, and upper gastrointestinal epithelium of sturgeon. Currently, iridovirus is recognized as the most predominant pathogenic virus affecting sturgeon. Clinical signs of infection include cessation of feeding, as well as edematous and pale gills (17). Electron microscopy confirmed that the characteristic cells contain large quantities of fully formed or developing virions of an icosahedral shape (18). These pathological changes result in respiratory issues for juvenile sturgeon and a deterioration of their overall health.

Hybrid sturgeon (*A. baerii*♀×*A. schrenckii*♂) is a species commonly bred in China, resulting from the hybridization of *A. baerii* and *A. schrenckii*. Compared with its parent species, this hybrid sturgeon has an increased reproductive capacity, growth rate, disease resistance, and survival rate, making it a popular choice among farmers and consumers (19). In August 2023, hybrid sturgeons cultured in Sichuan, China, exhibited pathological disease characterized by body surface ulcers, visceral bleeding, organ enlargement, and white nodules in the liver and spleen, resulting in a mortality rate exceeding 70%. To investigate the cause, pathogenic bacteria were isolated from the liver of diseased sturgeons and analyzed for their phenotypic and molecular traits. Furthermore, PCR and iridovirus-specific TaqMan real-time PCR (RT-PCR) analyses were conducted, revealing infection of *N. seriolae* and WSIV. Previous research has revealed coinfections of sturgeon with *Streptococcus iniae* and sturgeon herpesvirus-2, as well as cases of iridovirus infection (9, 20). However, this study presents the first documented case of *N. seriolae* infection in sturgeons.

We report the initial occurrence of *N. seriolae* and WSIV infection in sturgeon, examining the associated pathological manifestations and performing transcriptome sequencing to elucidate the impact of pathogen exposure and immune response profiles in infected sturgeon. The findings of this study provide a theoretical basis for understanding the pathogenic mechanisms of *Nocardia* and iridovirus infections, laying a theoretical foundation for the diagnosis and prevention of diseases in sturgeon.

## 2 Materials and methods

### 2.1 Clinical observation

Six diseased hybrid sturgeons (*A. baerii*♀×*A. schrenckii*♂), weighing 93.03 ± 4.14 g, were obtained from a sturgeon farm in Sichuan, China, while an additional six hybrid sturgeons from a different breeding base in Sichuan were selected as controls, matched in age and weight to the diseased fish. The control sturgeons exhibited normal behavior and feeding patterns, devoid of any visible physical injuries. Subsequently, all sturgeons were anesthetized with MS-222 for autopsy examination to investigate pathological changes and clinical characteristics.

### 2.2 Bacterial isolation and identification

Samples of infected fish were swabbed and sanitized with 75% ethanol. Specimens from the kidney, spleen, and liver were plated on brain heart infusion agar (BHIA) plates and then placed in a 26°C incubator for approximately 8 days. Single colonies were subsequently isolated using inoculation loops and transferred to fresh BHIA plates for purification. The pure colonies were characterized using Gram and acid-fast staining, along with PCR amplification targeting the *16S rRNA* gene to confirm the presence of *N. seriolae*. Bacteria from BHIA were transferred to a glass slide, spread evenly, and heat-fixed with a flame. Subsequently, Gram staining and acid-fast staining procedures were carried out. The air-dried slides were then observed under an oil immersion microscope. In Gram staining, positive bacteria are identified by a blue-violet color, whereas negative bacteria appear red. In acid-fast staining, a red color indicated a positive result, whereas a blue color indicated a negative result. In addition, bacterial genomic DNA was extracted using a Universal Genomic DNA Kit (CWBIO, Beijing, China) according to the manufacturer’s protocol and then stored at −20°C until use. The primers used in the research are detailed in **Supplementary Table S1**. PCR was carried out in a 50 μL mixture consisting of 25 μL of 2×Taq Plus MasterMix (CWBIO, Beijing, China), 0.2 μg of DNA, 2 μL of each primer (10 μM), and nuclease-free water to a total volume of 50 μL. The amplification process was carried out under the following conditions: initial denaturation at 94°C for 2 min; 35 cycles of denaturation at 94°C for 30 s, annealing at 55°C for 30 s, and extension at 72°C for 45 s; and a final extension step at 72°C for 2 min. The PCR products were visualized on a 1% agarose gel and subsequently sequenced by Sangon Biotech (Chengdu, China). The amino acid sequences of the isolated strains were analyzed using NCBI BLAST (http://blast.ncbi.nlm.nih.gov/Blast.cgi), and a phylogenetic tree was generated using the neighbor-joining method via MEGA 11 software.

### 2.3 TaqMan real-time PCR (RT-PCR) detection of WSIV virus

Spleen samples from infected hybrid sturgeons were utilized for genomic DNA extraction using a Universal Genomic DNA Kit (CWBIO, Beijing, China), in accordance with the manufacturer’s protocol. The genomic DNA extracted from the spleen samples was subsequently analyzed for the presence of WSIV by amplifying a consensus fragment of the major capsid protein (*MCP*) using TaqMan RT-PCR. Specific primers were synthesized for amplification of the WSIV *MCP* gene based on the methods described by Hofsoe-Oppermann et al. (21), forward primer (CSTCAACCRCGTGCCCRA), reverse primer (TCCCYGATGCGGACAAGT), TaqMan probe (FAM-CTTACTGGAYGCACCTATCTTCTCT-BHQ). TaqMan RT-PCR was performed with a LightCycler^®^ 480 real-time PCR system (Roche). Each reaction contained 12.5 μL of AceQ Universal U+ Probe Master Mix V2 (Vazymebiomed, Nanjing, China), 1 μL 10μM forward and reverse primers, 0.5μL 10μM TaqMan probe, 5 μL of nuclease-free water, and 5 μL of the template. The extracted DNA was used as the template for TaqMan RT-PCR, with both negative and positive controls (plasmid containing the target fragment) included in each reaction. The TaqMan RT-PCR program consisted of the following steps: 95°C for 10 min, followed by 45 amplification cycles at 95°C for 15 s, 60°C for 30 s, and 72°C for 45 s. The resulting data were analyzed using the LightCycler^®^ 480 real-time PCR system (Roche).

### 2.4 Histopathological examination

The microstructure and ultrastructure of hybrid sturgeons after bacterial and viral infection were analyzed using paraffin sectioning and transmission electron microscopy (TEM), and the healthy fish were used as the control. Tissue samples from the spleen, gill, liver, intestine, kidney, and splenic nodules were collected after MS222 anesthesia, fixed in 4% paraformaldehyde for 24 h, dehydrated in alcohol, cleared in xylene, and embedded in paraffin. Sections (5 μm thick) were obtained and subjected to hematoxylin and eosin (H&E) and acid-fast staining (Acid-Fast Stain Kit; Solarbio, Beijing, China). The sections were observed and photographed using a light microscope (DM500; Leica, Germany), and whole-slide imaging was performed with a Leica Microsystems DM1000 (Leica, Germany).

For TEM analysis, the tissues were fixed in 2.5% glutaraldehyde solution overnight at 4°C, followed by postfixation with 1% osmium tetroxide for 2 h. Dehydration was carried out using a series of acetone solutions with increasing concentrations (30%, 50%, 70%, 90%, and 100%). The samples were then infiltrated and embedded in Epox 812. Ultrathin sections, ranging from 60–90 nm in thickness, were sliced with a diamond knife, placed on metal grids, and double-contrasted with uranyl acetate and lead citrate. Finally, the sections were examined and photographed using a JEM-1400-FLASH transmission electron microscope (JEOL Ltd., Tokyo, Japan).

### 2.5 Transcriptome sequencing

Total RNA was extracted from the spleens of infected hybrid sturgeons using the TRIzol reagent (Invitrogen, Carlsbad, CA, USA) according to the manufacturer’s instructions, with healthy spleens serving as controls (n=3). The quantity and quality of the extracted RNA were evaluated through 1% agarose gel electrophoresis and an Agilent Bioanalyzer 2100 system (Agilent Technologies; Palo Alto, CA, USA). Subsequently, eukaryotic mRNA was enriched using oligo(dT) beads, whereas prokaryotic mRNA was enriched by removing rRNA with a Ribo-Zero™ Magnetic Kit (Epicenter, Madison, WI, USA). The enriched mRNA was then fragmented using fragmentation buffer and converted into cDNA with random primers. Second-strand cDNA synthesis was performed using DNA polymerase I, RNase H, and dNTPs. The cDNA fragments were ligated to Illumina sequencing adapters after the end-repair process. The products were purified and amplified and then sequenced by Gene Denovo Biotechnology Co. (Guangzhou, China) using the Illumina NovaSeq 6000 system (Illumina, San Diego, CA, USA).

### 2.6 Transcriptome data analysis

Adapter sequences, reads containing more than 10% unknown nucleotides, and low-quality reads (Q value ≤ 20) of the sequencing data were filtered using fastp to obtain clean reads (22). The clean reads were subsequently *de novo* assembled using Trinity software with default parameters (23). For functional annotation, the unigenes were compared with the NCBI nonredundant (NR) protein database (http://www.ncbi.nlm.nih.gov), Swiss-Prot database (http://www.expasy.ch/sprot), Kyoto Encyclopedia of Genes and Genomes (KEGG) database (http://www.genome.jp/kegg), and COG/KOG database (http://www.ncbi.nlm.nih.gov/COG) using the BLASTx program (http://www.ncbi.nlm.nih.gov/BLAST/) with an E-value threshold of 1e-5.

To determine the differential expression levels between the infected and control groups, RPKM values were utilized for normalizing gene expression levels using the RSEM and DESeq2 packages (24, 25). Genes with a false discovery rate (FDR) ≤ 0.05 and an absolute fold change ≥ 2 were identified as differentially expressed genes (DEGs). Gene Ontology (GO) functional enrichment and KEGG enrichment analyses of the DEGs were subsequently performed using the clusterProfiler package in R, and the results were visualized via the ggplot2 package (26).

To assess whether specific gene sets within pathways presented significant differences between control and diseased hybrid sturgeons, gene set enrichment analysis (GSEA) was performed using GSEA software and MSigDB (27). In summary, we input the gene expression matrix and ranked the genes via the SinaltoNoise normalization method. The enrichment scores and *P* values were calculated according to the default parameters. The Figdraw online platform (https://www.figdraw.com/) was employed to create schematic diagrams. Additionally, we analyzed the expression of genes related to the autophagy and mitophagy pathways and generated heatmaps.

### 2.7 Analysis validation of RNA-seq results

A total of 12 genes were randomly selected and evaluated via quantitative real-time PCR (qRT-PCR) with an ABI 7500 FAST real-time PCR system (Applied Biosystems, USA). The amplification reactions were performed with 2x SG Fast qPCR Master Mix (Sangon, Shanghai, China). The qRT-PCR conditions consisted of one denaturation cycle at 95°C for 3 min and 45 cycles of 15 s at 95°C, 30 s at an annealing temperature of 60°C, and 30 s at 72°C. The NormFinder program was used to evaluate the expression stability of the reference genes *β-actin*, glyceraldehyde-3-phosphate dehydrogenase (*GAPDH*), and eukaryotic translation elongation factor 1 alpha (*EF1α*) in accordance with the MIQE (Minimum information for publication of quantitative real-time PCR experiments) guidelines (28). Following this assessment, the relative expression of target genes in hybrid sturgeons was calculated using the 2^-ΔΔCt^ method (29). The primers employed for both the target and reference genes are detailed in **Supplementary Table S2**. Each assay was conducted in triplicate for every sample.

## 3 Results

### 3.1 Clinical symptoms of diseased hybrid sturgeons

**Figure 1:**
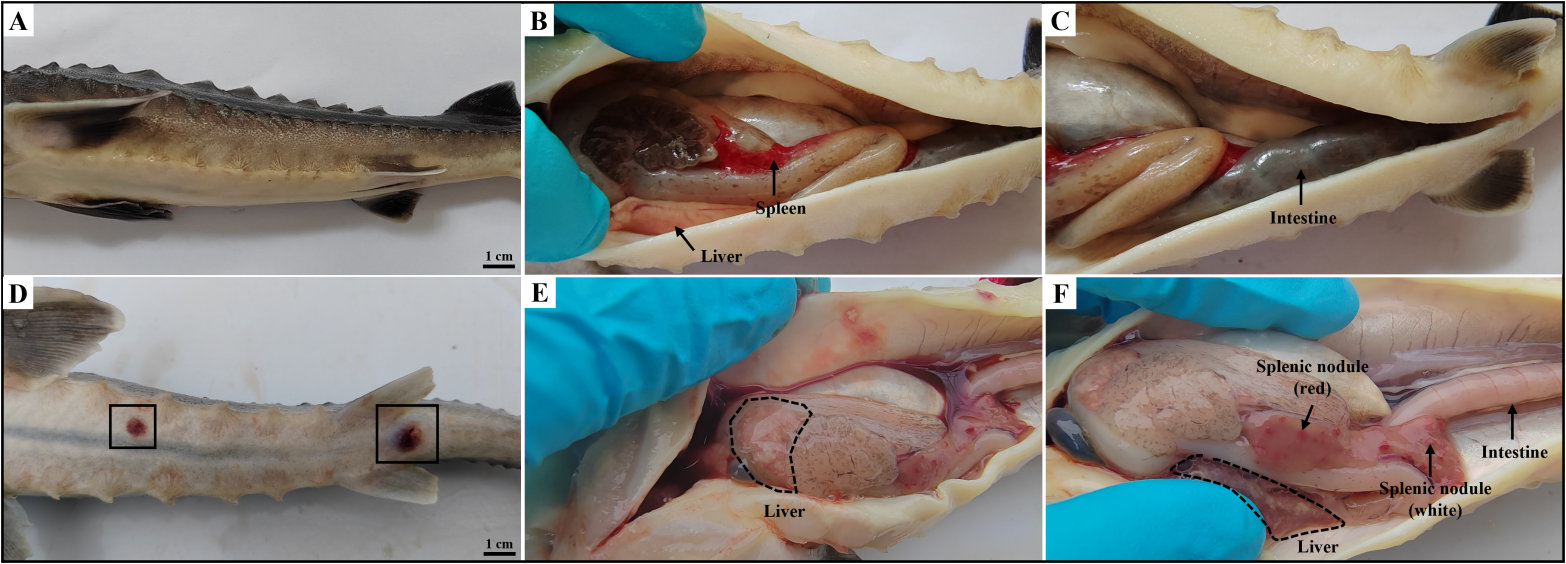
Clinical symptoms of diseased hybrid sturgeon. **(A–C)** Control hybrid sturgeon. **(D, E)** Diseased hybrid sturgeon. **(D)** Scattered ulcers (black box); **(E)** Liver whitening; **(F)** Numerous white or red nodules in spleen, and empty intestine.

The diseased hybrid sturgeons displayed typical symptoms such as sluggish swimming, delayed reactions, anorexia, and lethargy. Subsequent deaths occurred following the onset of symptoms, with a mortality rate as high as 70%. As shown in **Figure 1**, afflicted hybrid sturgeons presented diffuse hemorrhage in subcutaneous tissue, anal swelling, and bleeding. Additionally, scattered ulcers ranging from 0.5–1.0 cm in diameter were observed on the body and tip of the jaw. Anatomical observations revealed the presence of ascites and intestinal emptiness. The liver displayed signs of blanching, congestion, and hemorrhage, and the spleen was enlarged. Additionally, both the liver and spleen contained numerous nodules, either white or red, measuring approximately 0.1 to 0.5 cm.

### 3.2 Bacteriological examination

**Figure 2:**
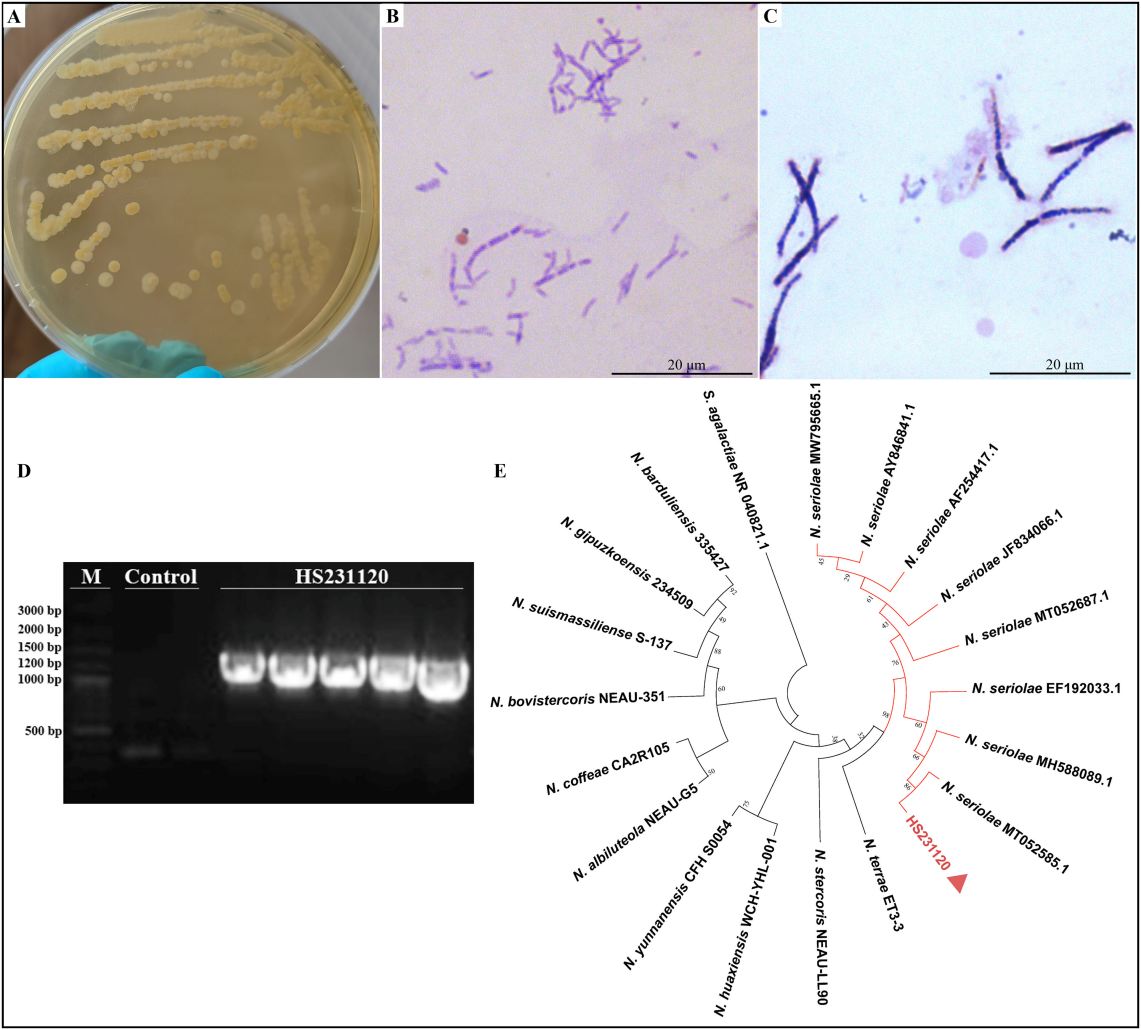
Morphology observation and phylogenetic tree based on sequences of *16S rRNA* gene of isolated *N. seriolae* (strain HS231120). **(A)** The colony morphology on BHIA plate; **(B)** Acid-fast staining; **(C)** Gram staining; **(D)** Electrophoresis of *16s rRNA* gene PCR of isolate HS231120; **(E)** Phylogenetic tree, and the red triangle represented the bacterium HS231120. Bootstrap values were displayed above each tree branch.

Cultures from the spleen and liver of hybrid sturgeons were inoculated into BHIA medium, resulting in the isolation and purification of a dominant strain, HS231120. The isolated pathogenic bacteria exhibited slow growth, taking 5–8 days to grow on BHIA medium. The colonies presented white or light yellow, dry, waxy, and wrinkled textures (**Figure 2A**). These bacteria were identified via gram-positive and weak acid-fast staining (**Figures 2B, C**). The bacteria appeared oval shaped or as long, slender rods with multiple septa, forming a well-defined mycelium that fragmented into irregular rod-shaped structures under high magnification. The amplification results of the *16S rRNA* gene from the isolated strain HS231120 are displayed in **Figure 2D**, revealing a distinct band at approximately 1300 bp. For the construction of a phylogenetic tree, *16S rRNA* gene sequences from the reference strains of *Nocardia* were selected from the GenBank database. The analysis revealed that isolate HS231120 clustered within the *N. seriolae* group (**Figure 2E**).

### 3.3 TaqMan RT-PCR detection of WSIV

**Figure 3:**
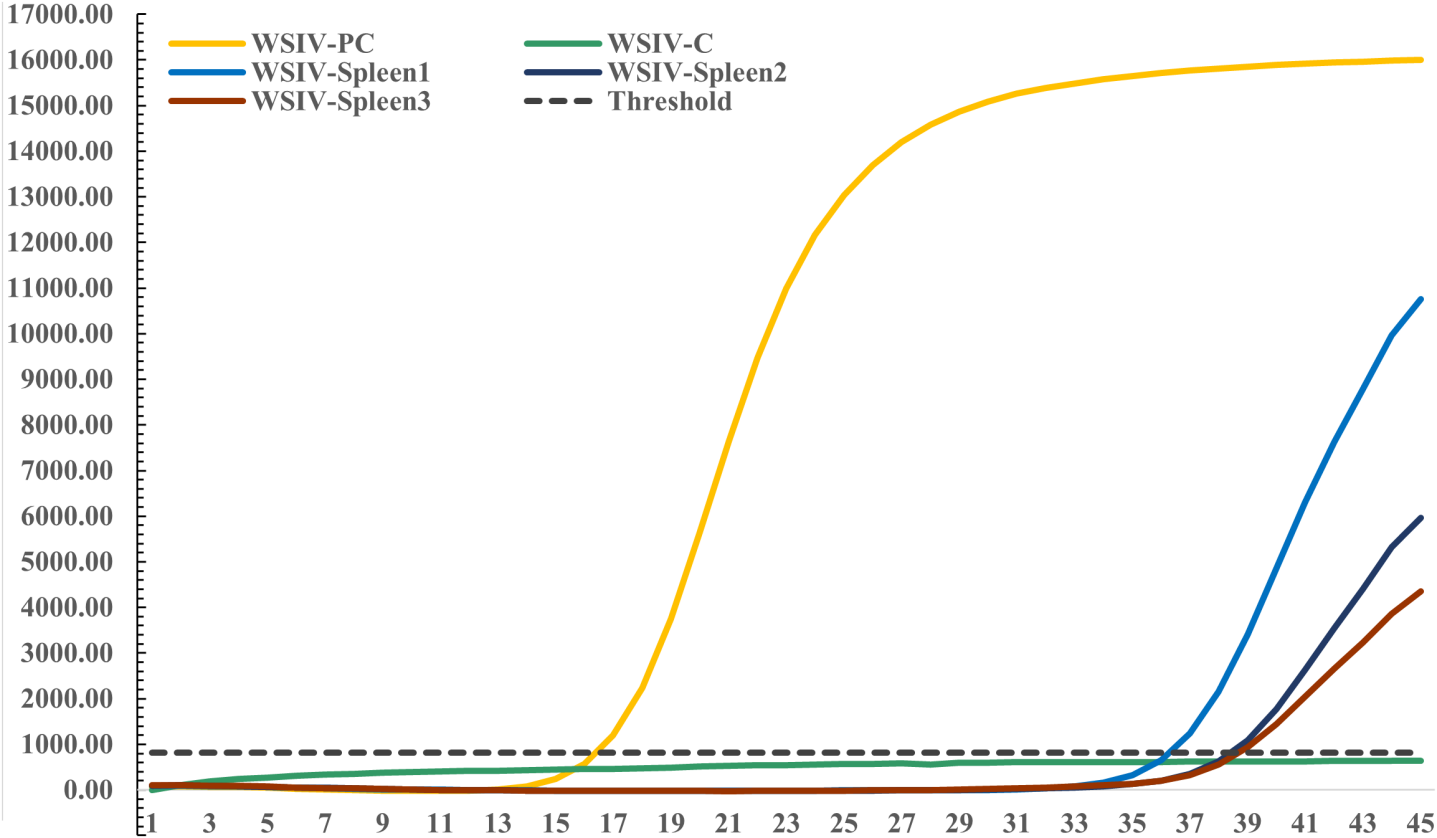
Amplification of the WSIV *MCP* gene using TaqMan RT-PCR.

Specific TaqMan RT-PCR detection targeting the WSIV *MCP* gene was conducted on spleen DNA extracted from diseased hybrid sturgeons. The results revealed a weak positive signal for WSIV in the spleen tissues from the diseased hybrid sturgeon. Additionally, the results revealed a clear amplification curve in the positive control, while the negative control did not show any amplification (**Figure 3**).

### 3.4 Histopathological characteristics

**Figure 4:**
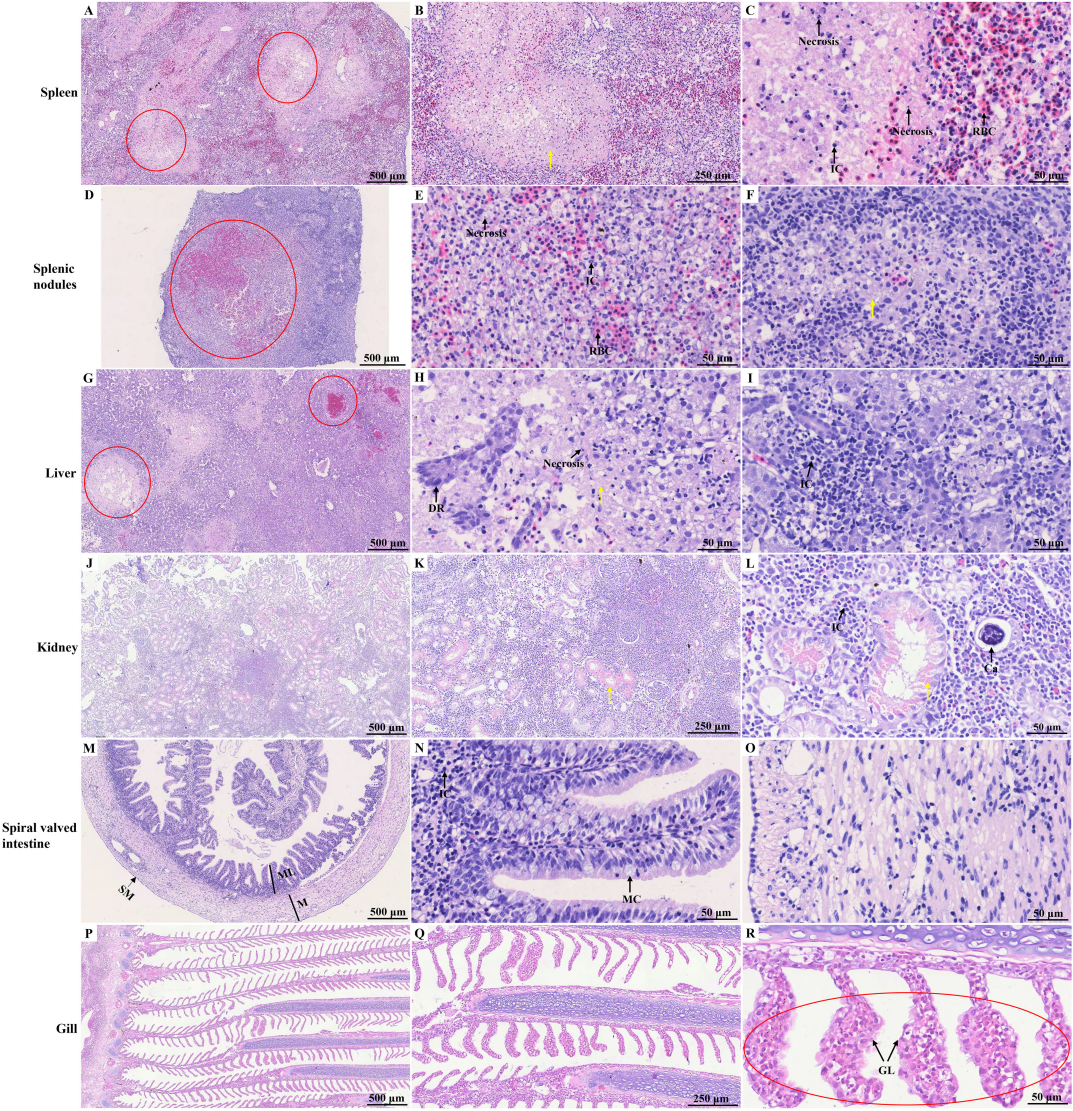
HE staining of different tissues of diseased hybrid sturgeon. **(A–C)** Spleen; **(D–F)** Splenic nodules; **(G–I)** Liver; **(J–L)** Kidney; **(M–O)** Spiral valved intestine; **(P–R)** Gill. The red circle highlights the site of significant pathological damage; the yellow arrow points to the homogeneous pink-stained exudate. Ca, calcification; DR, ductular reaction; GL, gill lamella; IC, inflammatory cell; M, muscular layer; MC, Mucous cell; ML, mucous layer; SM, serous membrane; RBC, red blood cell.

Histopathological analysis revealed no significant pathological damage in the tissues of control hybrid sturgeons (**Supplementary Figure S1**). HE staining of the spleen revealed indistinct red and white pulp structures with fading boundaries and multiple necrotic foci, indicating cell necrosis, inflammatory cell infiltration, and uniform pink-stained exudate (**Figures 4A–C**). Within the splenic nodules, a prominent necrotic focus was observed, accompanied by cell swelling, necrosis, inflammatory cell infiltration, and pink-stained exudate. Inflammatory edema was evident in the white pulp area, characterized by increased serous exudates that widened lymphocyte spaces and resulted in varying amounts of pink-stained exudates (**Figures 4D–F**). Necrotic lesions were identified in the diseased liver and were characterized by fragmented and concentrated nuclei within the necrotic hepatocytes. A variable amount of homogeneous pink-stained exudate surrounded these lesions. Inflammatory cell infiltrates, predominantly lymphocytes, were notably present in this region. Additionally, a local ductular reaction was observed (**Figures 4G–I**). In the kidney, local renal interstitial cells exhibited focal necrosis, characterized by cell necrosis and increased inflammatory cell infiltration. Exudates were occasionally observed in the renal tubules, along with transparent drop-like degeneration of renal tubular epithelial cells, as indicated by the presence of thick red-stained granules in the cytoplasm. Local calcification lesions were also identified (**Figures 4J–L**). The spiral valve intestine of hybrid sturgeons is organized into mucous, muscular, and serous layers from inner to outer. The mucous layer contained varying numbers of mucus cells. Histopathological analysis revealed no significant pathological damage in the diseased spiral valve intestine (**Figures 4M–O**). In the gill filaments, epithelial cell proliferation and thickening of the gill lamellae were observed (**Figures 4P–R**).

**Figure 5:**
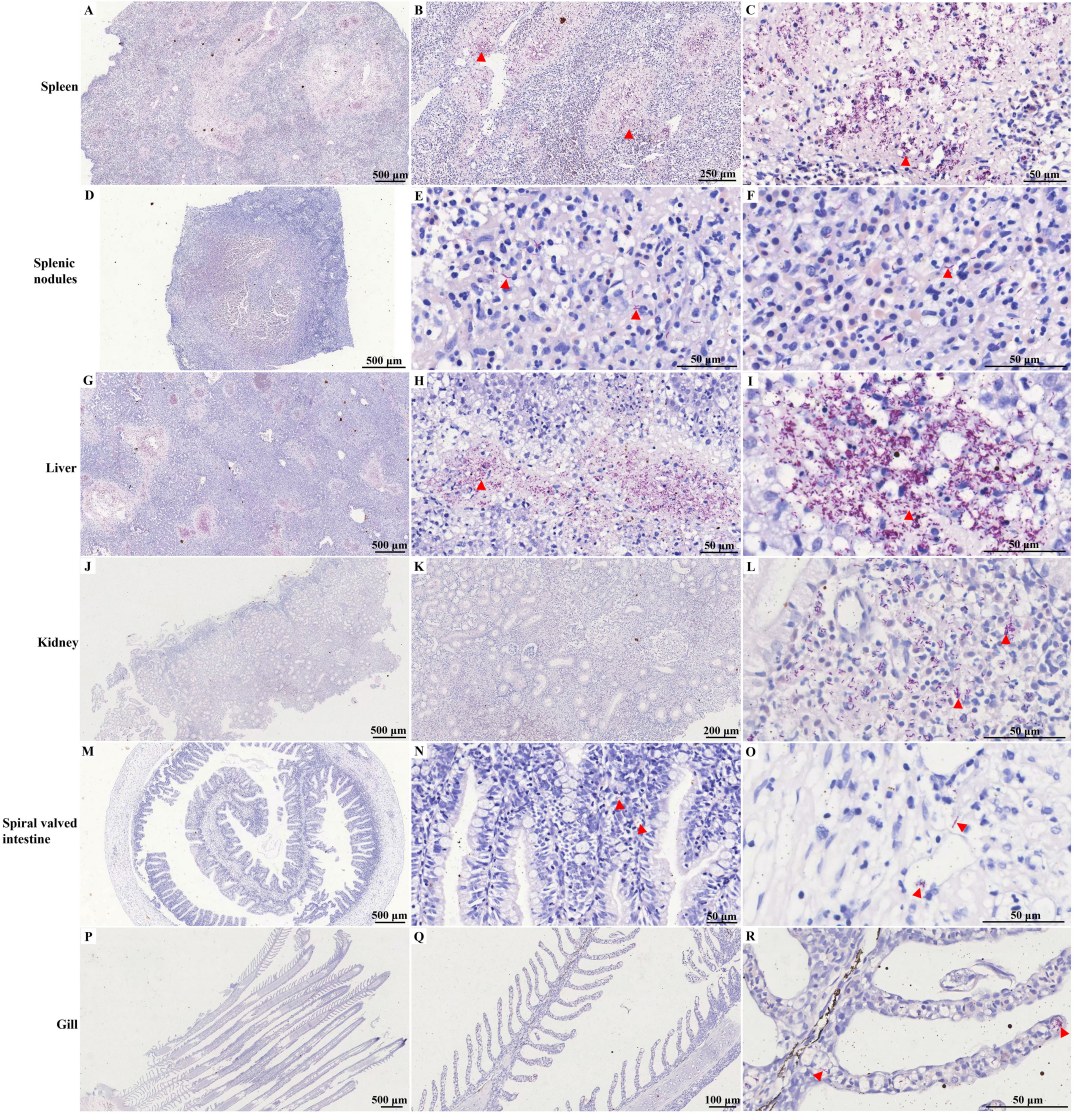
Acid-fast staining of different tissues of diseased hybrid sturgeon. **(A–C)** Spleen; **(D–F)** Splenic nodules; **(G–I)** Liver; **(J–L)** Kidney; **(M–O)** Spiral valved intestine; **(P–R)** Gill. The red triangle indicates weak positive staining for acid-fast bacteria.

As shown in **Figure 5**; **Supplementary Figure S2**, a notable presence of purple-red, weakly positive bacteria was observed in the spleen tissue, with sporadic occurrences identified in the splenic nodules through acid-fast staining. Clusters of these bacteria were also noted, accompanied by a significant amount of weakly positive purple-red staining signals, in the kidney. Additionally, the spiral valve intestine and gill presented sporadic weakly positive bacteria, as indicated by acid-fast staining.

### 3.5 TEM results

**Figure 6:**
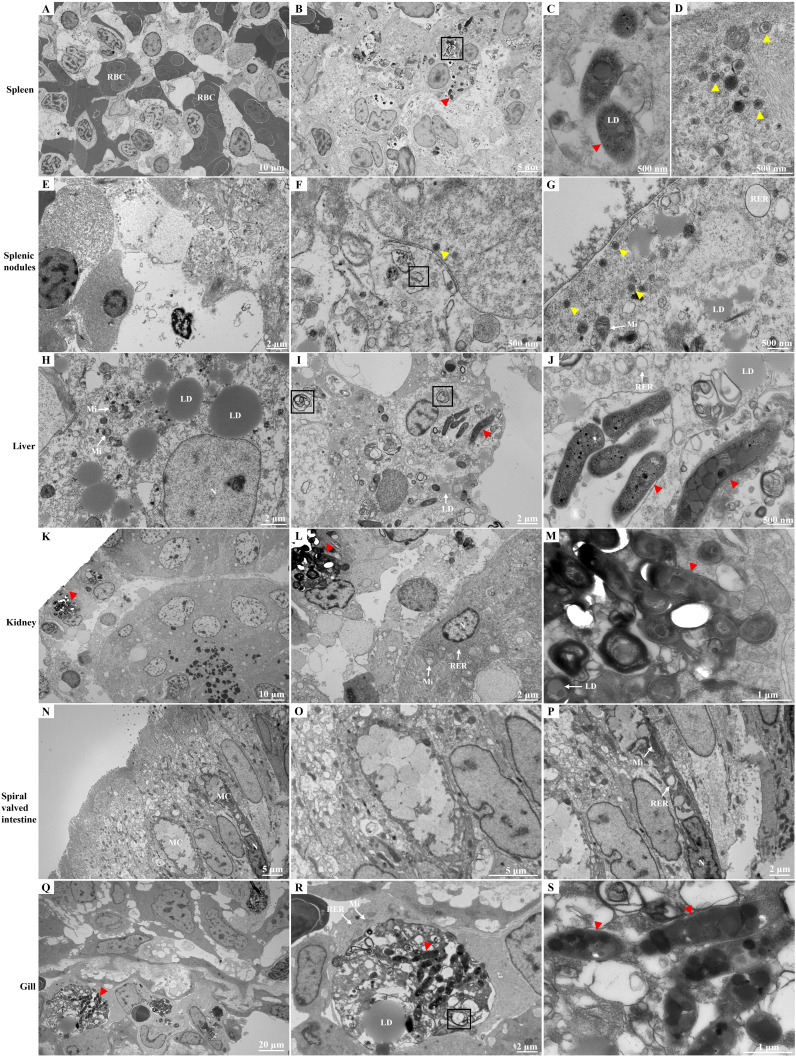
Ultrastructure of different tissues of diseased hybrid sturgeon. **(A–D)** Spleen; **(E–G)** Splenic nodules; **(H–J)** Liver; **(K–M)** Kidney; **(N–P)** Spiral valved intestine; **(Q–S)** Gill. The black box symbolizes autolysosome; the red triangle represents Nocardia; and the yellow triangle represents particles of iridovirus. LD, lipid droplet; MC, mucous cell; Mi, mitochondria; RBC, red blood cell; RER, rough endoplasmic reticulum.

The infected spleen tissue exhibited an abnormal morphological structure, with numerous red blood cells visible (**Figure 6A**). Long rod-shaped or club-shaped bacteria containing lipid droplets in their cytoplasm were observed, along with autophagy activity (**Figures 6B, C**). Virus particles measuring approximately 125–180 nm in diameter were also identified. These viral particles displayed a dense viral core with high electron density surrounded by membranes with low electron density (**Figure 6D**). Approximately hexagonal virus particles were also observed in the splenic nodules, with sizes similar to those observed in the infected spleen. Evidence of mitochondrial shrinkage, characterized by decreased mitochondrial cristae and increased membrane density, was noted. Additionally, noticeable expansion of the rough endoplasmic reticulum was observed (**Figures 6E–G**). Abnormal hepatocyte morphology and structure were identified. Hepatocyte nuclei exhibited an oval shape, with evenly distributed chromatin, which was primarily composed of euchromatin and heterochromatin located at the periphery. Mitochondrial swelling in the cytoplasm, dissolution and disruption of mitochondrial cristae, and loss of matrix were observed. Furthermore, expansion of the rough endoplasmic reticulum was noted, along with the presence of rod-shaped bacteria in the cytoplasm. Additionally, lipid droplets and autolysosomes were present (**Figures 6H–J**). In the kidney, a clustered distribution of bacteria was observed (**Figures 6K–M**). The morphological structure of mucous cells in the spiral valve intestine appeared slightly abnormal. The mitochondria in the mucous cell cytoplasm were relatively normal, whereas the rough endoplasmic reticulum was expanded, with no obvious bacteria observed (**Figures 6N–P**). Following infection, in epithelial cells in the gill, mitochondria exhibited a relatively normal morphological structure, along with an expanded rough endoplasmic reticulum. Numerous aggregated bacteria were visible in the cytoplasm of epithelial cells, as were multiple autolysosomes (**Figures 6Q–S**). No significant pathological damage in the tissues of control hybrid sturgeons by TEM (**Supplementary Figure S3**).

### 3.6 Transcriptome and functional annotation data

Transcriptome sequencing was performed on six cDNA libraries derived from the spleens of the control and infection groups, resulting in 38,259,562,200 raw reads (**Table 1**). After quality assessment and filtering, each library produced more than 5,929,909,005 clean reads. The Q30 values for all the samples exceeded 93%, with the GC content ranging from 42.83% to 45.61%, indicating high sequencing quality. A total of 86,970 unigenes, ranging from 201 bp to 17,567 bp in length, were identified, with an N50 length of 1,507 bp, using Trinity software. The raw sequencing data used in this study have been submitted to the Sequence Read Archive (SRA) database of NCBI (accession numbers SRR30204917- SRR30204922).

**Table 1:** Transcriptome sequencing and assembly information of spleen after infection in hybrid sturgeon.

Sample	Raw data (bp)	Clean data (bp)	Q20 rate (%)	Q30 rate (%)	GC (%)
C-Sp1	5,991,577,500	5,929,909,005	97.64	93.5	44.09
C-Sp2	6,568,880,400	6,501,875,448	97.81	93.74	45.61
C-Sp3	6,167,055,000	6,086,708,862	97.66	93.57	42.89
S-Sp1	6,103,513,500	6,012,737,948	97.51	93.25	42.86
S-Sp2	6,789,710,700	6,687,369,510	97.51	93.24	43.15
S-Sp3	6,638,825,100	6,526,207,321	97.73	93.75	42.83

**Figure 7:**
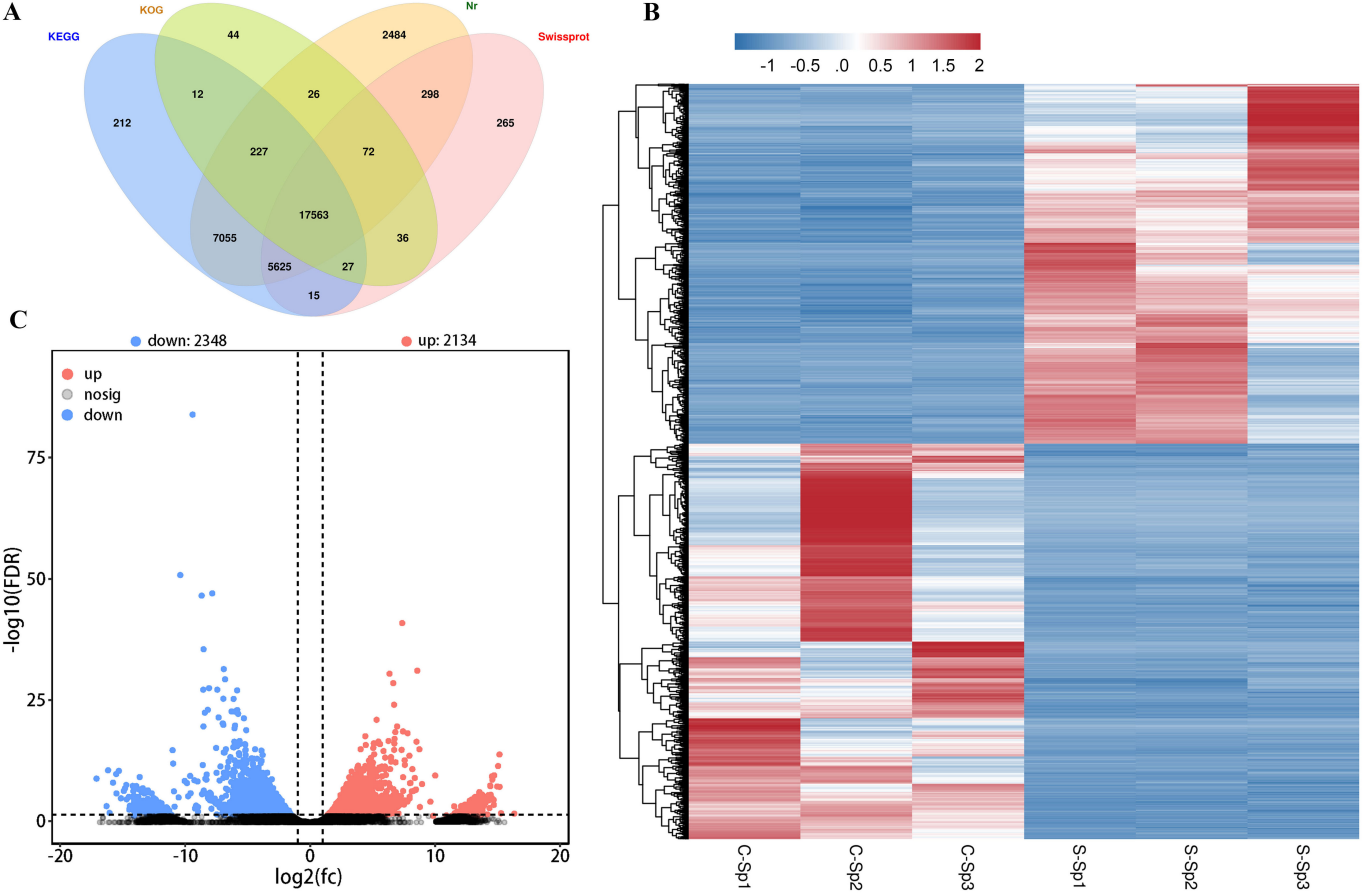
DGEs in spleen of the control and diseased hybrid sturgeons. **(A)** The Venn diagram of genes from hybrid sturgeon spleen against four databases (KEGG, KOG, NR, and Swissprot). **(B)** The heatmap of DEGs between the control and infection groups. **(C)** Volcano plot of DEGs between the control and infection groups. The blue dots represent down-regulation of gene expression, the red dots indicate up-regulation of gene expression, and the black dots signify genes with no significant differences in expression.

The assembled unigenes were aligned with sequences from 4 databases: 28,056 from KEGG, 15,634 from KOG, 29,585 from NR, and 21,244 from SwissProt (**Figure 7A**). Gene expression analysis was subsequently conducted using diseased hybrid sturgeons and control fish, revealing 4,482 DEGs (FDR ≤ 0.05, log2(fc) ≥ 2). A heatmap was used to visualize the levels of gene expression, with normalization performed through the z score to create hierarchical clusters of samples. Two distinct clusters emerged: one for genes downregulated after infection with *N. seriolae* and WSIV and another for genes upregulated (**Figure 7B**). The volcano plot revealed 2,134 significantly upregulated DEGs and 2,348 significantly downregulated DEGs following infection (**Figure 7C**).

### 3.7 GO and KEGG enrichment analyses

**Figure 8:**
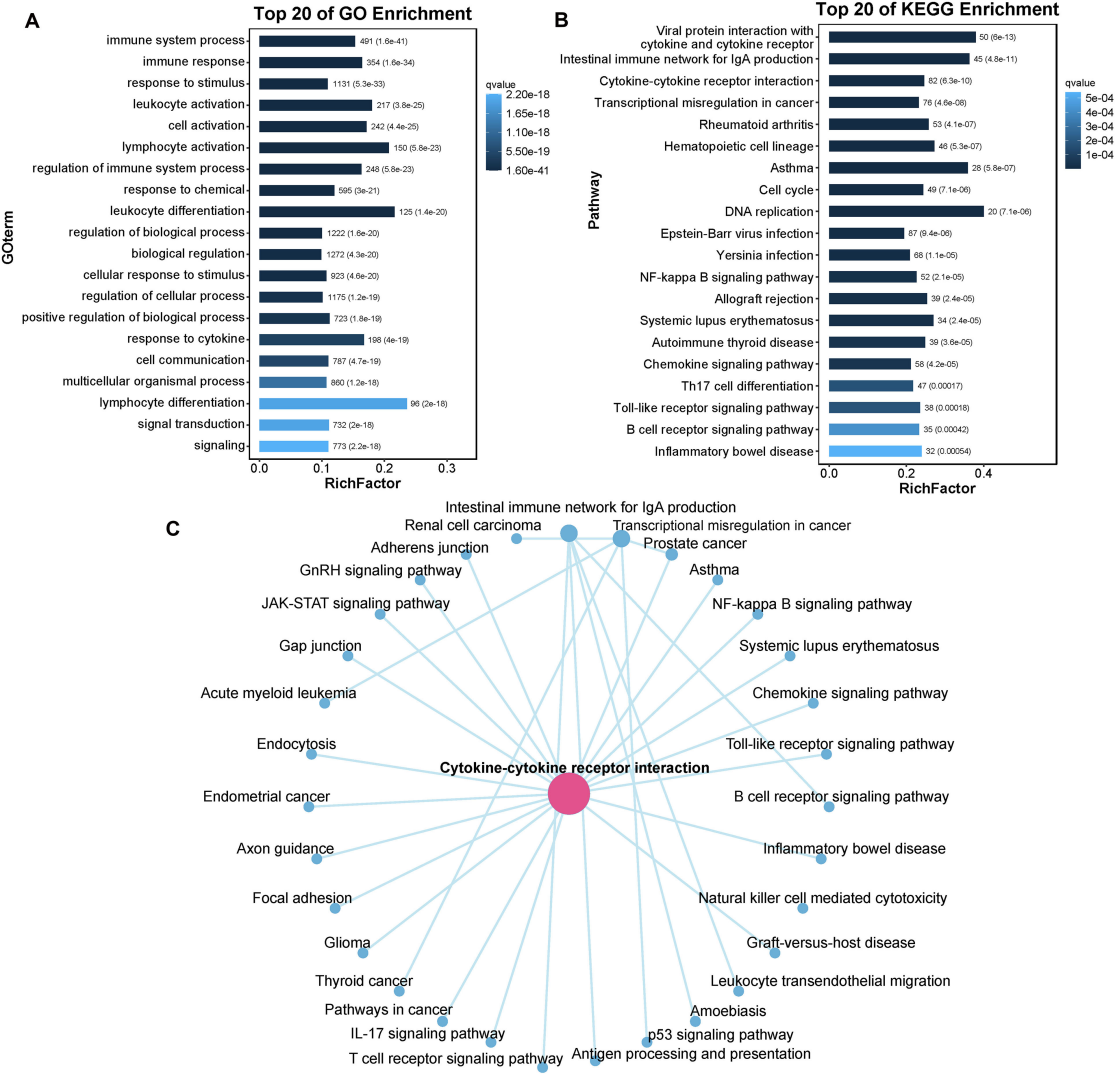
The enrichment of DEGs. **(A)** Barplot of the top 20 GO enriched terms for DEGs. **(B)** Barplot of the top 20 KEGG enriched pathways for DEGs. **(C)** The interaction network of KEGG enriched pathways. GO enrichment only concentrate on biological process (BP); RichFactor: the ratio of unigene number enriched in GO term or KEGG pathway to the annotated unigene number, indicating the degree of enrichment.

The DEGs were then subjected to GO term and KEGG pathway enrichment analyses. In the GO analysis, numerous DEGs between control and diseased individuals were found to participate in “immune system processes”, “immune responses”, “response to stimulus”, “leukocyte activation” and “cell activation” (**Figure 8A**). The KEGG pathway enrichment analysis revealed that the majority of DEGs were involved in pathways such as “Viral protein interaction with cytokine and cytokine receptor”, “Intestinal immune network for IgA production”, “Cytokine-cytokine receptor interaction”, and “Transcriptional misregulation in cancer” (**Figure 8B**). On the basis of the pathway enrichment information of the DEGs, we constructed a network diagram that illustrates the interaction relationships among various pathways, facilitating the identification of core pathways. The results indicated that the cytokine-cytokine receptor interaction exhibited greater connectivity with other pathways and served as a central component of the regulatory network (**Figure 8C**). In this study, a total of 45 DEGs were involved in the cytokine-cytokine receptor interaction signaling pathway, of which 12 DEGs were upregulated in diseased fish, including C-X-C motif chemokine ligand 5 (*CXCL5*), C-X-C motif chemokine ligand 8 (*CXCL8*), C-X-C motif chemokine receptor 2 (*CXCR2*), and interleukin 17 receptor c (*IL17RC*). The remaining 33 DEGs were downregulated in diseased fish, including C-C motif chemokine ligand 14 (*CCL14*), C-C motif chemokine receptor 3 (*CCR3*), and C-C motif chemokine receptor 4 (*CCR4*). The detailed expression data are presented in **Supplementary Table S3**.

**Figure 9:**
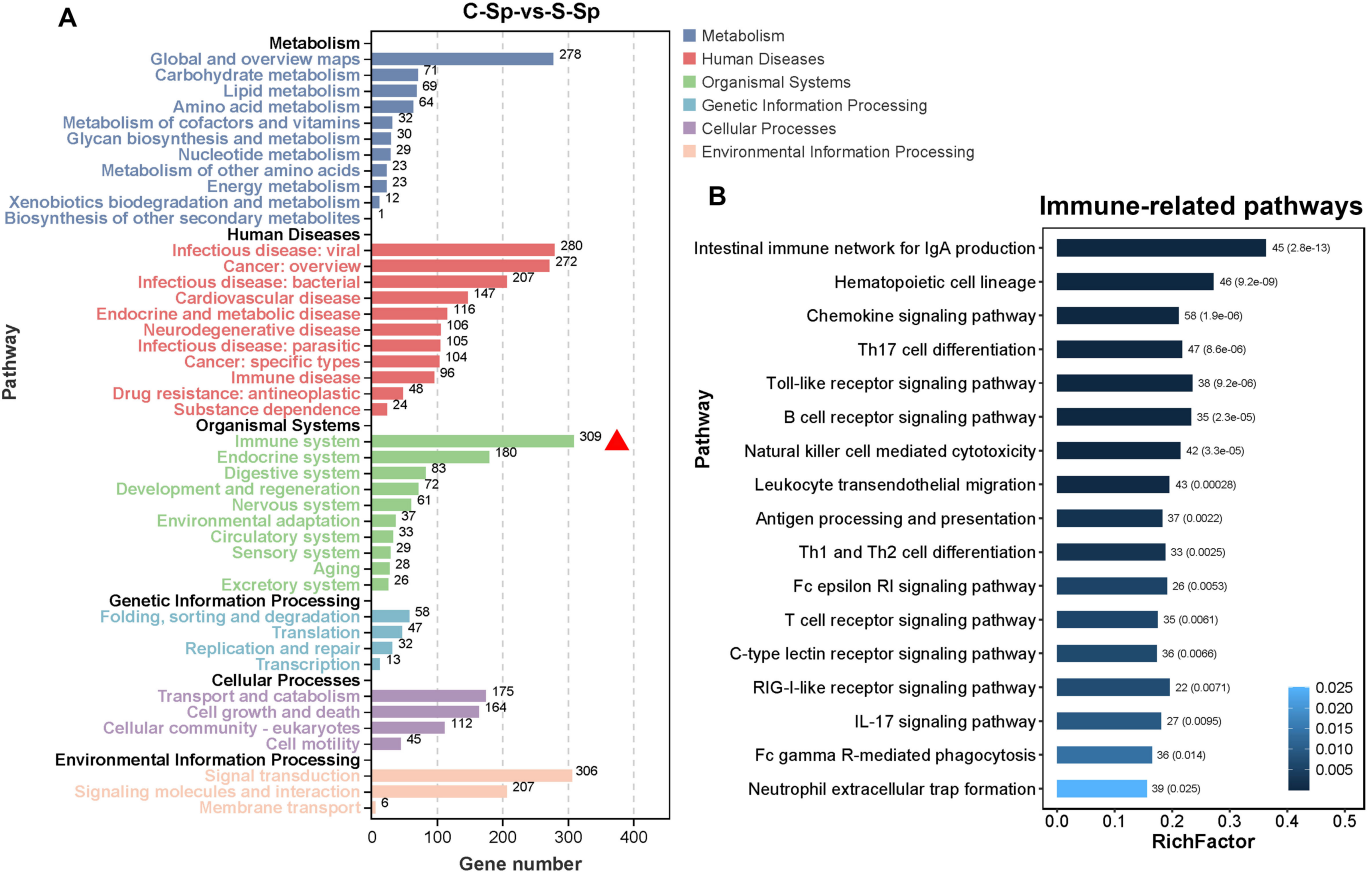
Immune system subcategory analysis. **(A)** The number of DEGs within the subcategory of the KEGG pathway. The red triangle represents the immune system subcategory. **(B)** The pathway information enriched within the immune system subcategory.

We subsequently quantified the number of DEGs within subcategories of KEGG pathways. As illustrated in **Figure 9A**, the immune system subcategory presented the greatest number of DEGs. A total of 309 DEGs were significantly enriched across 17 immune-related signaling pathways, including “Intestinal immune network for IgA production”, “Chemokine signaling pathway”, “Th17 cell differentiation”, “Toll-like receptor signaling pathway”, and “B cell receptor signaling pathway” (**Figure 9B**). The expression profiles of several immune-related DEGs are listed in **Supplementary Table S4**. For example, the expression of several genes that play crucial roles in the immune system, such as T-cell-specific surface glycoprotein CD28 (*CD28*), T-cell surface glycoprotein CD8 alpha chain (*CD8A*), T cell-specific protein-tyrosine kinase (*LCK*), zeta chain of T cell receptor associated protein kinase 70 (*ZAP70*), tyrosine-protein kinase BTK (*BTK*), B-cell receptor CD22 (*CD22*), CD79 alpha (*CD79A*), and perforin 1(*PEF1*), was significantly downregulated after infection.

### 3.8 GSEA-based KEGG pathway enrichment

**Figure 10:**
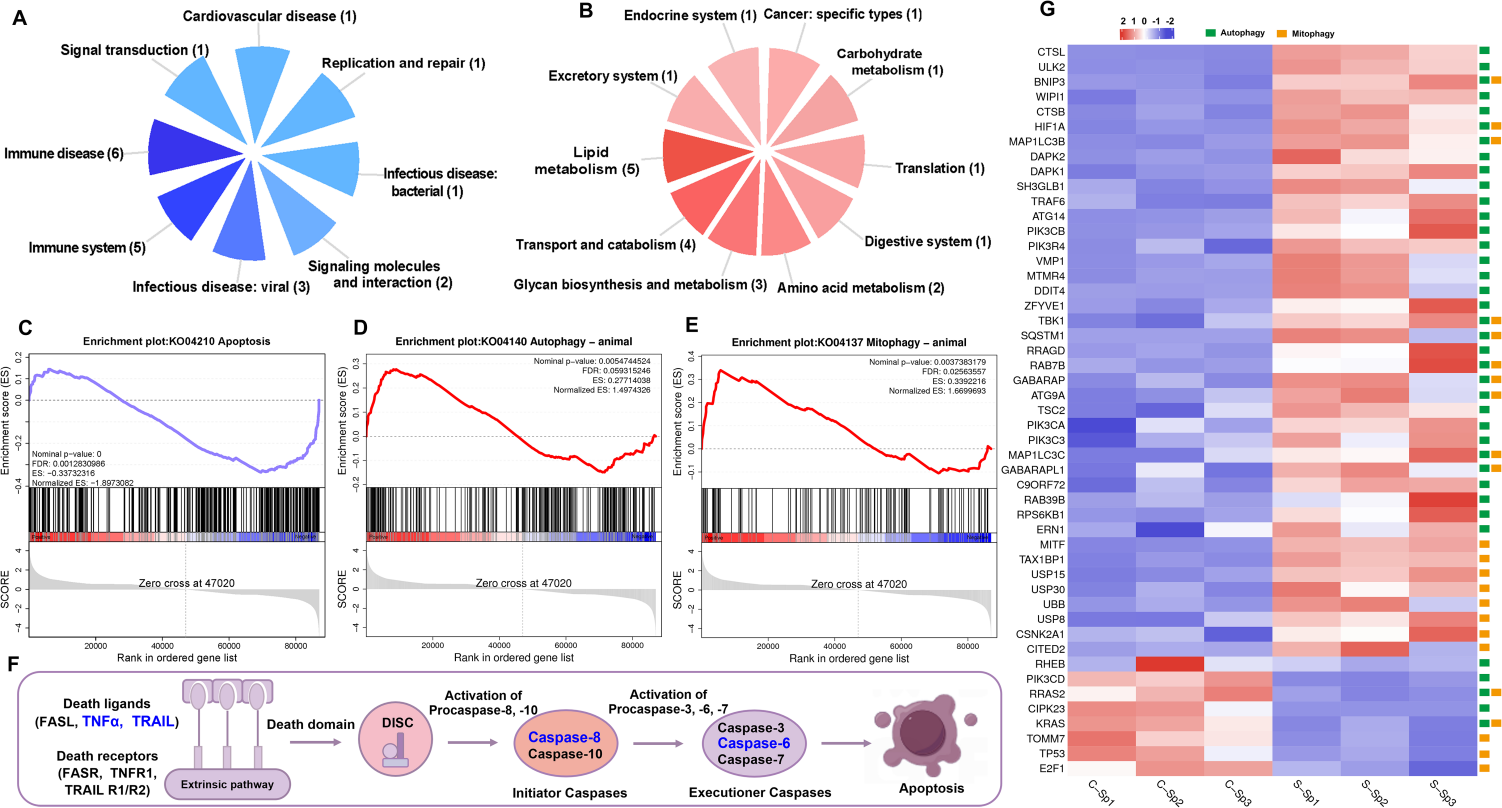
GSEA analysis of down- and up-regulated genes after *N. seriolae* and WSIV co-infection. **(A)** The distribution of subcategories among the top 20 enriched pathways of down-regulated genes. **(B)** The distribution of subcategories among the top 20 enriched pathways of up-regulated genes. **(C–E)** Snapshot of GSEA enrichment plots including apoptosis signaling pathway (down-regulated), autophagy and mitophagy signaling pathway (up-regulated). The normalized enrichment score (NES) reflected the results of the analysis across different gene sets. The nominal *p*-value indicated the significance of enrichment for each specific gene set. **(F)** A schematic diagram of the death receptor-mediated apoptosis pathway, with genes that exhibit significantly down-regulated expression highlighted in blue font. **(G)** The heatmap of selected genes in autophagy and mitophagy signaling pathway. Green boxes represent genes involved in the autophagy pathway, while yellow boxes indicate genes associated with the mitophagy pathway.

GSEA was employed to assess the enrichment of signaling pathways in the control and infection groups. Overall, the downregulated genes were enriched predominantly in categories related to immune diseases and the immune system, whereas the upregulated genes were associated primarily with categories pertaining to lipid metabolism (**Figures 10A, B**). Notably, the apoptosis signaling pathway was significantly suppressed, whereas the autophagy and mitophagy signaling pathways were activated (**Figures 10C–E**). The expression of genes within the death receptor-mediated apoptosis pathway revealed that tumor necrosis factor alpha (*TNFα*) and TNF superfamily member 10 (*TRAIL*), along with caspase-6 (*CASP6*) and caspase-8 (*CASP8*), were significantly downregulated, resulting in the inhibition of apoptosis (**Figure 10F**). Most identified genes in the autophagy and mitophagy signaling pathways, such as unc-51 like autophagy activating kinase 2 (*ULK2*), BCL2 Interacting Protein 3 (*BNIP3*), WD repeat domain phosphoinositide-interacting protein 1 (*WIPI1*) and cathepsin B (*CTSB*), presented significantly upregulated expression profiles following infection. Conversely, the expression of the tumor protein P53 (*TP53*) gene was significantly downregulated after infection (**Figure 10G**).

### 3.9 qRT-PCR verification

**Figure 11:**
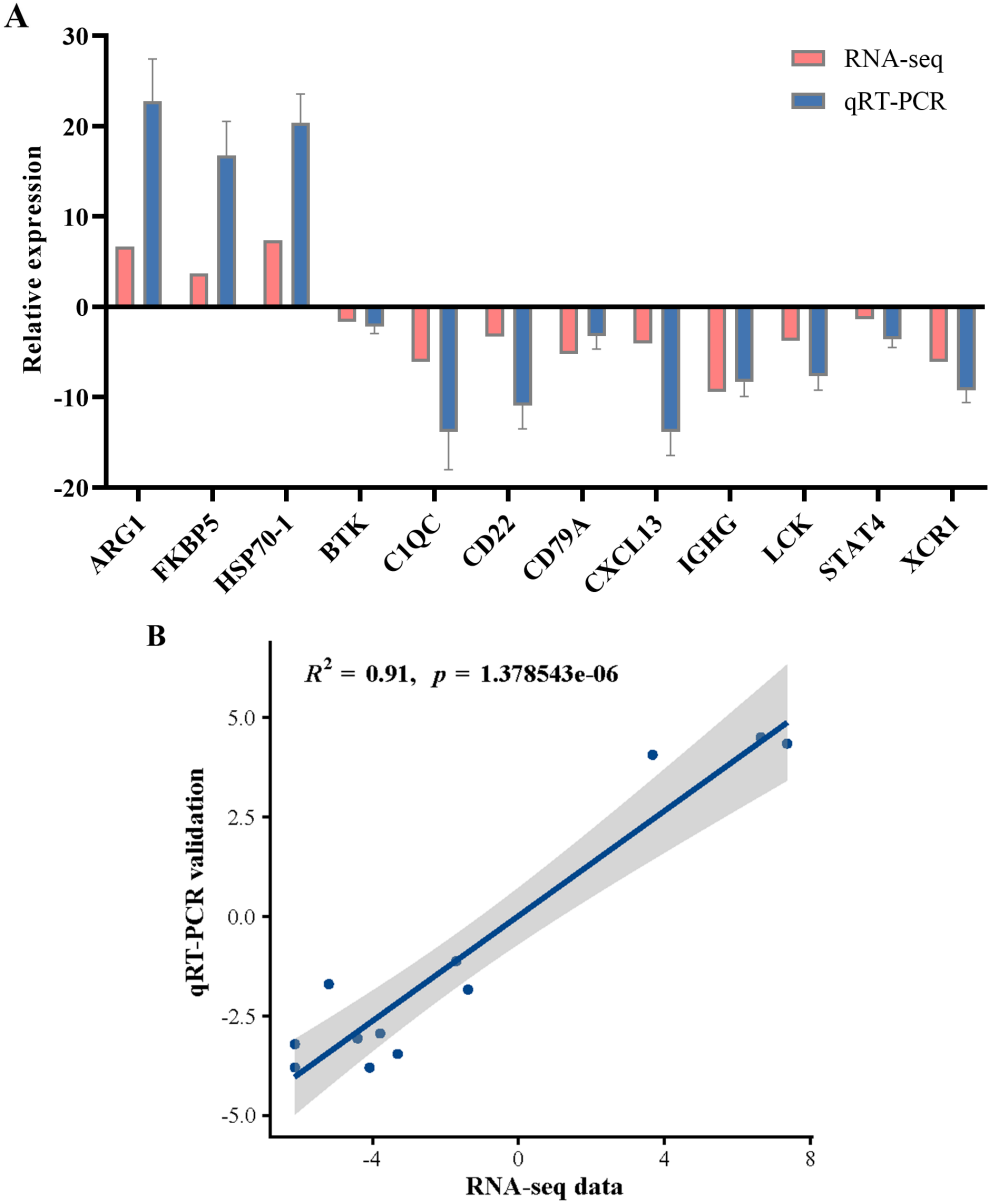
qRT-PCR validation of the selected genes for RNA-seq data. **(A)** Comparison of RNA-seq and qRT-PCR results. qRT-PCR data were presented as the means ± SEM (n=3). **(B)** Scatterplot of the correlation coefficient between qRT-PCR and RNA-seq.

To validate the DEGs identified through RNA-seq, 12 genes from diverse categories were selected for qRT-PCR confirmation. In accordance with the MIQE guidelines, we employed the NormFinder program to assess the expression stability of the internal reference genes *β-actin*, *GAPDH*, and *EF1α*. The stability values for *β-actin*, *GAPDH*, and *EF1α* were 0.207, 0.891, and 1.305, respectively. The results indicated that *β-actin* demonstrated superior stability, leading to its selection as the internal reference for data normalization in this study. The fold changes measured via qRT-PCR were subsequently compared with the RNA-seq expression data. As shown in **Figure 11**, the qRT-PCR results were significantly correlated with the RNA-seq results, suggesting concordance with the results of the transcription analysis.

## 4 Discussion

In aquaculture, the widespread cultivation of sturgeon is attributed to its rapid growth rate and resistance to diseases (3, 30). However, the rise of large-scale and intensive fish farms has led to frequent and severe disease outbreaks in sturgeons, significantly impeding their cultivation (31). Recent reports have highlighted the substantial economic losses caused by *N. seriolae* or iridovirus infections in aquaculture (16, 32). This case represents the initial documented occurrence of Nocardiosis in sturgeon, along with the first reported instance of infection involving both *N. seriolae* and iridovirus, leading to notable economic losses. Despite the severe impact, the pathogenic mechanisms by which *N. seriolae* and iridovirus affect hybrid sturgeons remain elusive. Here, we investigated the immune response of hybrid sturgeons during infection with *N. seriolae* and iridovirus.

*N. seriolae* is a filamentous, partially acid-fast and gram-positive bacterium that causes nocardiosis in marine and freshwater fish, such as the largemouth bass (*Micropterus salmoides*) (23), large yellow croaker (*Larimichthys crocea*) (33), cultured eel (*Anguilla japonica*) (34) and northern snakehead (*Channa argus*) (35). Nocardiosis in fish typically has a prolonged onset and is characterized by anal enlargement, ascites, and the formation of multiple white nodules in the liver, kidney, and spleen. In this study, the diseased hybrid sturgeons initially presented symptoms such as anorexia, weight loss, reduced swimming activity, and, in some cases, backstroke behavior. As the disease progresses, punctate ulcers gradually develop on the body surface. Autopsy revealed numerous white or red nodules in the spleen and liver. *Nocardia* was successfully isolated from the kidney and spleen of the affected fish, and its morphology and staining properties were consistent with those of *Nocardia* observed in previously reported cases of fish nocardiosis, which resulted in similar pathological changes (34). Phylogenetic analyses of the *16S rRNA* gene confirmed that isolate HS231120 clustered within the *N. seriolae* group. In summary, the morphological, staining, physiological, and biological characteristics of this isolate from diseased hybrid sturgeons support its classification as *N. seriolae*.

Research has demonstrated that viral diseases significantly contribute to mass mortality in sturgeon populations. Sturgeon iridoviruses, such as WSIV, are the primary pathogens responsible for mortality in cultured white sturgeon in North America and Russian sturgeon in Europe (36). Iridoviruses can also infect various commercial fish species, including rock bream (37), large yellow croaker (38), and grouper (39). Affected fish typically exhibit diffuse hemorrhage in the subcutaneous tissue, with the most pronounced symptoms including spleen and kidney enlargement, gallbladder enlargement, bleeding spots in the gill filaments, and pale anemia in the liver. In this case, the TaqMan RT-PCR analysis of WSIV in the spleen tissue of the diseased hybrid sturgeons yielded a weakly positive result. Additionally, iridovirus-like particles measuring 125 to 180 nm in diameter, along with long rod-shaped bacteria, were observed in tissue via electron microscopy. In this study, we observed that the clinical features and pathological lesions of infected hybrid sturgeons presented characteristics consistent with both *N. seriolae* and iridovirus infections. These findings, in conjunction with the bacteriological results, confirm that infection with *N. seriolae* and WSIV was the cause of this disease in hybrid sturgeons. However, the TaqMan RT-PCR results were only weakly positive, indicating that further investigation is necessary to determine whether this finding is associated with the virulence of WSIV. Additionally, the isolation of the virus requires the use of a sturgeon-derived cell line. Despite the existence of various sturgeon cell lines, our current unavailability of these resources hindered the successful isolation of iridovirus in this study, posing challenges for future research. In addition, the simultaneous infection by two pathogens resulted in extensive tissue necrosis, a shortened disease course, and a wide range of lesions. Consequently, the necrotic nodules we observed may represent the early stages of granuloma formation, occurring prior to the development of typical granuloma structures, ultimately leading to widespread mortality among the affected fish.

In aquaculture, infection caused by *N. seriolae* is a protracted process. This study revealed that the mortality rate resulting from the combined infections of *N. seriolae* and WSIV can reach as high as 70%, which exceeds the 30% mortality rate associated with N. seriolae infection alone (40, 41). To dissect the immune response of hybrid sturgeons to *N. seriolae* and WSIV infection, spleen transcriptomes were analyzed. A total of 4,482 DEGs were identified, and comparisons between the infected hybrid sturgeons and the control sturgeons revealed a greater number of downregulated genes than upregulated genes. These findings suggest that *N. seriolae* and WSIV infection primarily suppresses gene expression, potentially leading to disruptions in biological functions. These results are consistent with previous transcriptome studies conducted on *Paralichthys olivaceus* infected with viral hemorrhagic septicaemia virus (VHSV) (42). In contrast, following infection with *N. seriolae*, hybrid snakehead (*Channa maculate*♀× *Channa argus*♂) presented a greater number of upregulated genes than significantly downregulated genes (40). This disparity indicated that the host response to infections caused by different bacteria or viruses varies considerably.

The DEGs were subsequently investigates through GO and KEGG pathway enrichment analyses. GO analysis revealed that several DEGs were involved in multiple immune-related subcategories, predominantly including “Immune system processes,” “Immune responses,” and “Response to stimulus.” This suggested that the immune system of hybrid sturgeons significantly changed in response to *N. seriolae* and WSIV infection. Concurrently, the KEGG pathway analysis revealed that cytokine-cytokine receptor interactions exhibited greater connectivity with other pathways and that the majority of these DEGs were associated with immune-related pathways. Notably, the immune system subcategory presented the greatest number of DEGs, with 309 DEGs significantly enriched across 17 immune-related signaling pathways, including the “Intestinal immune network for IgA production”, “Toll-like receptor signaling pathway”, “B cell receptor signaling pathway”, and “T cell receptor signaling pathway”. This finding suggested that host immunity was altered following infection, thereby contributing to resistance against pathogens. Pathways in the immune system are also altered by pathogenic infections in various cultured fish species. Our findings aligned with prior research showing changes in immune-related pathways in fish species like *Micropterus salmoides*, *Oreochromis niloticus*, and *C. argus* following exposure to pathogens (43, 44). These findings suggest that essential immune system pathways in teleosts are conserved and activated in response to pathogenic threats.

Cytokines, which are soluble proteins or glycoproteins found outside cells, serve as crucial regulators and mobilizers of the cells involved in innate immune responses. They also play significant roles in the adaptive inflammatory immune system and the restoration of homeostasis (45). In this study, a total of 45 DEGs were involved in the core pathway—cytokine-cytokine receptor interaction signaling pathways—and the expression of 12 DEGs, including *CXCL5*, *CXCL8*, *CXCR2*, and *IL17RC*, was upregulated in diseased fish. The expression of the remaining 33 DEGs, including *CCL14*, *CCR3*, and *CCR4*, was downregulated in diseased fish. These findings are similar to those reported in the spleen transcriptomes of hybrid snakehead affected by nocardiosis disease and suggest that infections caused by *N. seriolae* and WSIV disrupt the cytokine-cytokine receptor interaction mechanism in the spleens of hybrid sturgeons. The expression profiles suggest that different components of *N. seriolae* and WSIV might regulate TLRs, suggesting that several TLR-mediated signaling pathways could concurrently participate in the immune response to pathogen infection. The innate immune response plays a crucial role in combating pathogens and providing protection against infections during the initial phases. If pathogens breach these defense mechanisms, antigen-presenting cells (APCs, including dendritic cells, macrophages, and B lymphocytes) subsequently initiate a specific or adaptive immune response (46). *Nocardia* infection is characterized by distinct granulomatous inflammation, representing a distinctive pattern of chronic inflammatory reactions (47). This type of inflammation is typically observed in response to persistent or difficult-to-clear pathogens, such as *Nocardia*. This complex process involves the interaction of host humoral and cellular elements with antigens of the pathogen.

During the early phase of infection, various components of innate immunity play fundamental roles. Moreover, B lymphocytes and T lymphocytes are pivotal in orchestrating humoral and cellular immune responses against various pathogens, such as viruses, bacteria, and parasites. Upon recognition of an antigen presented by primary major histocompatibility complex (MHC) molecules, the TCR pathway is activated. This activation, along with its downstream signaling cascades, is crucial for immune responses. In this study, we identified genes related to the TCR pathway, including *CD28*, *CD8A*, *LCK*, and *ZAP70*, which were significantly downregulated postinfection. Similar findings have been reported in other teleost fishes (48, 49). *CD28* plays a crucial role in T cell activation, triggering cell proliferation and cytokine production and enhancing T cell survival. The CD8 antigen functions as a coreceptor alongside the T cell receptor on T lymphocytes, facilitating the identification of antigens presented by antigen-presenting cells within the context of class I MHC molecules (50). *LCK* serves as an essential signaling molecule during T cell selection and development. *ZAP70* encodes an enzyme belonging to the protein tyrosine kinase family that plays a significant role in T cell development and lymphocyte activation. When the antigens presented on MHC molecules bind to TCRα/β, LCK and ZAP70 are activated, leading to the initiation of downstream signaling cascades (51). The BCR pathway is essential for the activation and differentiation of B cells, which play crucial roles in the adaptive immune response (52). This signaling pathway facilitates the recognition of specific antigens and the subsequent production of antibodies that target and neutralize pathogens. In the present study, we identified the expression profiles of key immune genes related to the BCR signaling pathway, including *BTK*, *CD22*, *CD79A*, and *CD79B*. Notably, the expression of these genes was significantly downregulated after infection. Both *BTK* and *CD22* are essential for B cell development and activation (53). Additionally, *CD79A* functions in conjunction with *CD79B* to initiate the signal transduction cascade triggered by the binding of antigens to the B cell antigen receptor complex, leading to the internalization of the complex, trafficking to late endosomes, and antigen presentation (54). Overall, our results indicated that the expression of many members of the TCR and BCR signaling pathways, including the costimulatory molecules *ZAP70*, *BTK*, and *CD79A*, was significantly downregulated, suggesting that the TCR and BCR signaling pathways, which are critical for cellular immunity, may be temporarily inhibited following infection. However, the exact mechanism underlying this inhibition remains unclear.

Additionally, we observed the significant downregulation of *PRF1* gene expression following infection. This finding is consistent with previous results obtained after infectious spleen and kidney necrosis virus (ISKNV) infection in mandarin fish (*Siniperca chuatsi*) (55). The *PRF1* gene encodes a protein that forms pores and is critical for granzyme-induced programmed cell death and for protecting against cells infected by viruses or affected by neoplasia (56). A reduction in the level of the perforin protein may hinder the entry of effector proteins, thereby diminishing their capacity to facilitate programmed cell death in target cells infected by *N. seriolae* and WSIV. Conversely, the expression of the heat shock 70 kDa protein 1A (*HSP70-1*) gene, which plays an important role in the response to external environmental stress, was significantly upregulated following infection. This finding indicates a complex interplay between the pathogen and the host that influences the immune response during bacterial and viral infections.

Recent studies have revealed that infection and immunity involve distinct ‘dynamic games’ between viruses and hosts, wherein apoptosis plays a crucial role (57). In *P. olivaceus* infected with lymphocystis disease virus (LCDV), the formation of lymphocystis cells occurred primarily through the suppression of apoptosis, which involved the downregulation of the expression of genes such as *casp3*, *casp6*, and *casp8*, along with several other apoptosis-inducing genes (58). In our study, GSEA revealed that the apoptosis signaling pathway was significantly suppressed. Gene expression within the death receptor-mediated apoptosis pathway revealed that the expression of *TNFα* and *TRAIL*, along with that of *CASP6* and *CASP8*, was significantly downregulated, resulting in the inhibition of apoptosis and assisting the virus in escaping the host antiviral response. Furthermore, GSEA demonstrated that the autophagy and mitophagy signaling pathways were activated after infection. Many genes in the autophagy and mitophagy signaling pathways, such as *ULK2*, *BNIP3*, *WIPI1* and *CTSB*, presented significantly upregulated expression profiles following infection. Conversely, the expression of the *TP53* gene was significantly downregulated after infection. Generally, autophagy inhibits the induction of apoptosis, whereas the activation of apoptosis-related caspases suppresses the autophagy process (59). Studies have demonstrated that autophagy can selectively eliminate activated Caspase 8, thereby inhibiting tumor necrosis factor-related apoptosis-inducing ligand (TRAIL)-induced apoptosis (60). Our findings corroborate this finding, as the results of this study indicated a significant upregulation in the expression of autophagy-related genes, whereas the expression of *TRAIL*, *CASP8*, and *CASP6* was notably downregulated. The p53 signaling pathway has dual effects on the viral response by inhibiting innate antiviral immunity and apoptosis in virus-infected cells. This inhibition promotes viral replication in WSIV-infected cells and facilitates the transmission of newly formed viral particles to neighboring cells. Viruses have developed various strategies to inhibit apoptosis during infection, thereby facilitating their own replication; however, the precise mechanisms involved in infection in fish have yet to be elucidated.

## 5 Conclusion

This study examined hybrid sturgeons exhibiting signs of natural infection with *Nocardia* and iridovirus in an aquaculture pond. Through *16S rRNA* sequencing, *N. seriolae* was confirmed as the causative agent of nocardiosis, while TaqMan RT-PCR targeting the *MCP* gene identified the iridovirus as WSIV. Histopathological evaluations revealed significant damage across various tissues and organs in the affected hybrid sturgeons. Notable observations included long rod-shaped or club-shaped bacteria containing lipid droplets and viral particles. The transcriptomic profiles of the spleens from both infected and control hybrid sturgeons were analyzed via RNA-seq, resulting in the identification of 4,482 DEGs in the infected fish. GO and KEGG analyses highlighted multiple immune-related genes and pathways, suggesting that several biological processes—such as Toll-like receptor signaling pathways, B cell receptor signaling pathways, T cell receptor signaling pathways, cytokine-cytokine receptor interactions, apoptosis, autophagy, and mitophagy—are likely critical to the host immune response to infection with *N. seriolae* and WSIV. Our findings provide essential data for future studies on the immunogenetics of hybrid sturgeons and offer insights into the pathogenic mechanisms associated with bacterial–viral infections, potentially informing prevention and treatment strategies for *N. seriolae* and iridovirus in fish.

## Data Availability

The data presented in the study are deposited in the Sequence Read Archive (SRA) database of NCBI repository, accession number SRR30204917- SRR30204922.

## Supplementary material

SUPPLEMENTARY Table S1 Primers for PCR used in this study.

SUPPLEMENTARY Table S2 Gene-specific primers for qRT-PCR.

SUPPLEMENTARY Table S3 DEGs in the cytokine-cytokine receptor interaction signaling pathway.

SUPPLEMENTARY Table S4 Several immune-related DEGs regulated after infection.

SUPPLEMENTARY Figure S1 HE staining of different tissues of control hybrid sturgeon. **(A–C)** Spleen; **(D–F)** Liver; **(G–I)** Kidney; **(J–L)** Spiral valved intestine; **(M–O)** Gill. CM, circular muscle; GF, gill filament; GL, gill lamella; He, hepatocyte; LM, longitudinal muscle; M, muscular layer; MC, Mucous cell; ML, mucous layer; SM, serous membrane; RBC, red blood cell.

SUPPLEMENTARY Figure S2 Acid-fast staining of different tissues of control hybrid sturgeon. **(A–C)** Spleen; **(D–F)** Liver; **(G–I)** Kidney; **(J–L)** Spiral valved intestine; **(M–O)** Gill.

SUPPLEMENTARY Figure S3 Ultrastructure of different tissues of control hybrid sturgeon. **(A, B)** Spleen; **(C, D)** Liver; **(E, F)** Kidney; **(G, H)** Spiral valved intestine; **(I, J)** Gill. LD, lipid droplet; Lym. lymphocyte; MC, mucous cell; Mi, mitochondria.
