# Supplementary material for: Natural infection of hybrid sturgeon (Acipenser baerii♀×Acipenser schrenckii♂) with Nocardia seriolae and white sturgeon iridovirus: pathological and transcriptomic analyses
Source: Front Immunol. 2024 Nov 22;15:1488159. doi: 10.3389/fimmu.2024.1488159 (PMC11621106; doi:10.3389/fimmu.2024.1488159)
Supplement: SUPPLEMENTARY Table S1 — Primers for PCR used in this study. [file DataSheet1.docx]

| **Table S1** Primers for PCR used in this study | |
| --- | --- |
| **Name** | **Sequence (5′ to 3′)** |
| 27F | AGAGTTTGATCMTGGCTCAG |
| 1492R | GGTTACCTTGTTACGACTT |

| **Table S2** Gene-specific primers for qRT-PCR | |  |
| --- | --- | --- |
| **Gene** | **Sequence (5′ to 3′)** | **Product (bp)** |
| *ARG1*-F | ACACTGTGAAGAAGGAAGGAAGG | 157 |
| *ARG1*-R | GTTGGAGATGTCAGCGGAGTG |  |
| *FKBP5*-F | TCACCTTCAACCTTGGCAGAG | 111 |
| *FKBP5*-R | CGTAGGCGTACTCCGAATGG |  |
| *HSP70*-F | GACCGCAGGAGGAGTTATGAC | 91 |
| *HSP70*-R | GTTGTCCGAGTAGGTGGTGAAG |  |
| *BTK*-F | TCCACTACCATCAACACAACTCC | 199 |
| *BTK*-R | TGCTTGCCTTGCCACTTCC |  |
| *C1QC*-F | CACCAACACAGAGGACCACTAC | 121 |
| *C1QC*-R | GCTCATCAGACTCAGGCACAG |  |
| *CD22*-F | GCTACACCTGGTATGGGAATGG | 123 |
| *CD22*-R | CAACCTCGTTCTTAACTTCACACTC |  |
| *CD79A*-F | AGTTCTCTCACGGGACCTACC | 212 |
| *CD79A*-R | ATTCTCCTCCTCTTCCTTGTTGTAC |  |
| *CXCL13*-F | CGCTGTGGCAAGGTGGAG | 124 |
| *CXCL13*-R | GCTCGTTGGTGTTTCTTCTCTG |  |
| *IGHG*-F | CACTAAGGAAGAATGGCTGGAATC | 144 |
| *IGHG*-R | TCAACAGATGGAGGCGATATGC |  |
| *LCK*-F | AGATAGTTACATACGGTCGGATTCC | 209 |
| *LCK*-R | GGTGGCTGTGAAGAAGTCCTC |  |
| *STAT4*-F | GACTGCCTCATTCTCCTCACTC | 225 |
| *STAT4*-R | TCTCTAAGCACTTGGTCGGTATG |  |
| *XCR1*-F | GTTGTTATCTTCTTACGGACACTGG | 225 |
| *XCR1*-R | TGGCTGCTCTTCGGTATTCTTATAG |  |
| *β-actin*-F | CGTGCGTGACATTAAGGAGAAG | 176 |
| *β-actin*-R | GGAAGGAAGGCTGGAAGAGTG |  |

| **Table S3** DEGs in the cytokine-cytokine receptor interaction signaling pathway | | | |  |  |
| --- | --- | --- | --- | --- | --- |
| **Name** | **Description** | **log2(fc)** | **Change** |  |  |
| *BMP2* | Bone morphogenetic protein 2 | 9.62 | Up |  |  |
| *BMP7* | Bone morphogenetic protein 7 | 3.52 | Up |  |  |
| *CXCL5* | C-X-C motif chemokine ligand 5 | 2.00 | Up |  |  |
| *CXCL8* | C-X-C motif chemokine ligand 8 | 3.12 | Up |  |  |
| *CXCR2* | C-X-C motif chemokine receptor 2 | 3.03 | Up |  |  |
| *IL17RC* | Interleukin 17 receptor c | 3.34 | Up |  |  |
| *IL18RAP* | Interleukin 18 receptor accessory protein | 4.04 | Up |  |  |
| *IL1R2* | Interleukin 1 receptor type 2 | 6.48 | Up |  |  |
| *LEPR* | Leptin receptor | 2.72 | Up |  |  |
| *MPL* | Myeloproliferative leukemia protein | 3.01 | Up |  |  |
| *TNFRSF11A* | TNF receptor superfamily member 11a | 1.90 | Up |  |  |
| *TNFRSF6B* | TNF receptor superfamily member 6b | 3.87 | Up |  |  |
| *ACKR4* | Atypical chemokine receptor 4 | -2.37 | Down |  |  |
| *CCL4* | C-C motif chemokine ligand 4 | -5.22 | Down |  |  |
| *CCL14* | C-C motif chemokine ligand 14 | -3.60 | Down |  |  |
| *CCL24* | C-C motif chemokine ligand 24 | -4.05 | Down |  |  |
| *CCR2* | C-C motif chemokine receptor 2 | -3.08 | Down |  |  |
| *CCR3* | C-C motif chemokine receptor 3 | -4.64 | Down |  |  |
| *CCR4* | C-C motif chemokine receptor 4 | -4.40 | Down |  |  |
| *CCR7* | C-C motif chemokine receptor 7 | -6.71 | Down |  |  |
| *CCR9* | C-C motif chemokine receptor 9 | -2.47 | Down |  |  |
| *CNTFR* | Ciliary neurotrophic factor receptor | -3.42 | Down |  |  |
| *CXCL1* | C-X-C motif chemokine ligand 1 | -4.52 | Down |  |  |
| *CXCL11* | C-X-C motif chemokine ligand 11 | -4.47 | Down |  |  |
| *CXCL12* | C-X-C motif chemokine ligand 12 | -3.80 | Down |  |  |
| *CXCL13* | C-X-C motif chemokine ligand 13 | -4.08 | Down |  |  |
| *CXCR1* | C-X-C motif chemokine receptor 1 | -2.48 | Down |  |  |
| *CXCR3* | C-X-C motif chemokine receptor 3 | -4.33 | Down |  |  |
| *CXCR4* | C-X-C motif chemokine receptor 4 | -2.39 | Down |  |  |
| *CXCR5* | C-X-C motif chemokine receptor 5 | -4.72 | Down |  |  |
| *EDAR* | Ectodysplasin a receptor | -6.69 | Down |  |  |
| *GDF6A* | Growth differentiation factor 6a | -2.35 | Down |  |  |
| *GH1* | Growth hormone 1 | -8.14 | Down |  |  |
| *IL12RB2* | Interleukin 12 receptor subunit beta 2 | -2.95 | Down |  |  |
| *IL13RA1* | Interleukin 13 receptor subunit alpha 1 | -1.87 | Down |  |  |
| *IL22RA2* | Interleukin 22 receptor subunit alpha 2 | -12.78 | Down |  |  |
| *IL2RB* | Interleukin 2 receptor subunit beta | -3.98 | Down |  |  |
| *LEP* | Leptin | -5.46 | Down |  |  |
| *LTBR* | Lymphotoxin beta receptor | -2.62 | Down |  |  |
| *TGFBR2* | Transforming growth factor beta receptor 2 | -5.52 | Down |  |  |
| *TNF* | Tumor necrosis factor | -2.83 | Down |  |  |
| *TNFRSF11B* | TNF receptor superfamily member 11b | -4.24 | Down |  |  |
| *TNFRSF9* | TNF receptor superfamily member 9 | -3.06 | Down |  |  |
| *TNFRSF19* | TNF receptor superfamily member 19 | -3.27 | Down |  |  |
| *XCR1* | X-C motif chemokine receptor 1 | -6.13 | Down |  |  |

| **Table S4** Several immune-related DEGs regulated after infection. | | | |  |  |  |
| --- | --- | --- | --- | --- | --- | --- |
| **Name** | **Description** | **log2(fc)** | **Change** | | | **Pathway** |
| *CD28* | T-cell-specific surface glycoprotein CD28 | -2.05 | Down | | | T cell receptor signaling |
| *CD8A* | T-cell surface glycoprotein CD8 alpha chain | -2.19 | Down | | | T cell receptor signaling |
| *LCK* | T cell-specific protein-tyrosine kinase | -3.79 | Down | | | T cell receptor signaling |
| *ZAP70* | Zeta chain of T cell receptor associated protein kinase 70 | -1.35 | Down | | | T cell receptor signaling |
| *TRB* | T cell receptor beta locus | -3.01 | Down | | | T cell receptor signaling |
| *CD3E* | CD3 epsilon subunit of T-cell receptor complex | -2.89 | Down | | | T cell receptor signaling |
| *CD247* | T-cell surface glycoprotein CD3 zeta chain | -2.90 | Down | | | T cell receptor signaling |
| *GRAP2* | GRB2 related adaptor protein 2 | -4.74 | Down | | | T cell receptor signaling |
| *ITK* | IL2 inducible T cell kinase | -3.32 | Down | | | T cell receptor signaling |
| *MAPK13* | Mitogen-activated protein kinase 13 | -1.84 | Down | | | T cell receptor signaling |
| *AKT1* | RAC-alpha serine/threonine-protein kinase | -2.25 | Down | | | T/B cell receptor signaling |
| *PIK3CB* | Serine/threonine protein kinase PIK3CB | 1.94 | Up | | | T/B cell receptor signaling |
| *BTK* | Tyrosine-protein kinase BTK | -1.70 | Down | | | B cell receptor signaling |
| *CD22* | B-cell receptor CD22 | -3.31 | Down | | | B cell receptor signaling |
| *CD79A* | CD79 alpha | -5.20 | Down | | | B cell receptor signaling |
| *CD79B* | CD79 beta | -7.90 | Down | | | B cell receptor signaling |
| *CD81* | CD81 molecule | -3.48 | Down | | | B cell receptor signaling |
| *IGHM* | Immunoglobulin mu chain | -6.15 | Down | | | B cell receptor signaling |
| *IGHV3-21* | Immunoglobulin heavy variable 3-21 | -14.19 | Down | | | B cell receptor signaling |
| *IGHV3-49* | Immunoglobulin heavy variable 3-49 | -11.01 | Down | | | B cell receptor signaling |
| *IGHV3-66* | Immunoglobulin heavy variable 3-66 | -13.84 | Down | | | B cell receptor signaling |
| *RAC2* | Rac family small GTPase 2 | -2.22 | Down | | | B cell receptor signaling |
| *RASGRP3* | RAS guanyl releasing protein 3 | 3.12 | Up | | | B cell receptor signaling |
| *SYK* | Tyrosine-protein kinase SYK | -1.51 | Down | | | B cell receptor signaling |
| *HSP70-1* | Heat shock 70 KDa protein 1A | 7.37 | Up | | | Antigen processing and presentation |
| *PRF1* | Perforin 1 | -6.91 | Down | | | Natural killer cell mediated cytotoxicity |


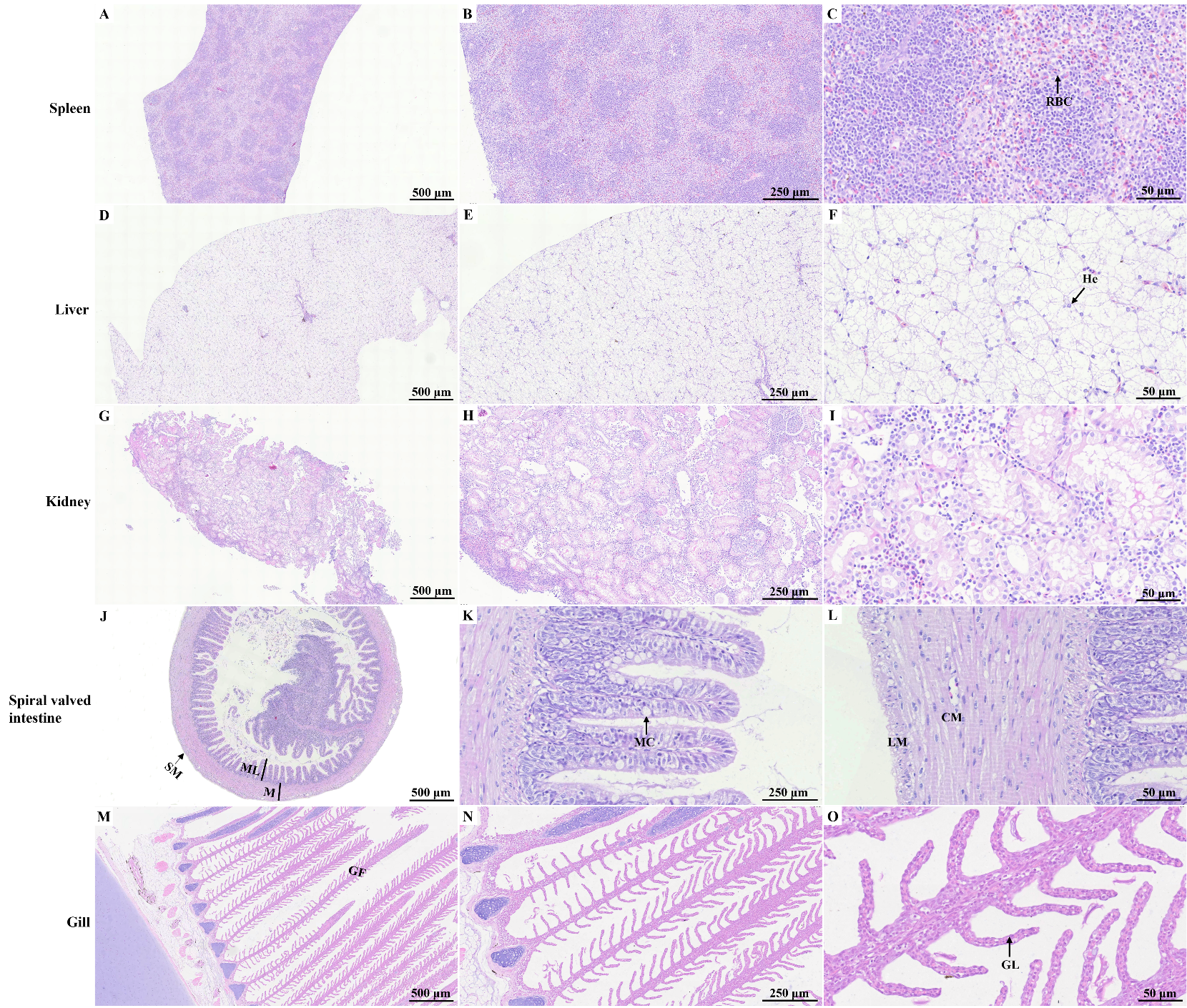
**Figure S1** HE staining of different tissues of control hybrid sturgeon. (A-C) Spleen; (D-F) Liver; (G-I) Kidney; (J-L) Spiral valved intestine; (M-O) Gill. CM. circular muscle; GF. gill filament; GL. gill lamella; He. hepatocyte; LM. longitudinal muscle; M. muscular layer; MC. Mucous cell; ML. mucous layer; SM. serous membrane; RBC. red blood cell.


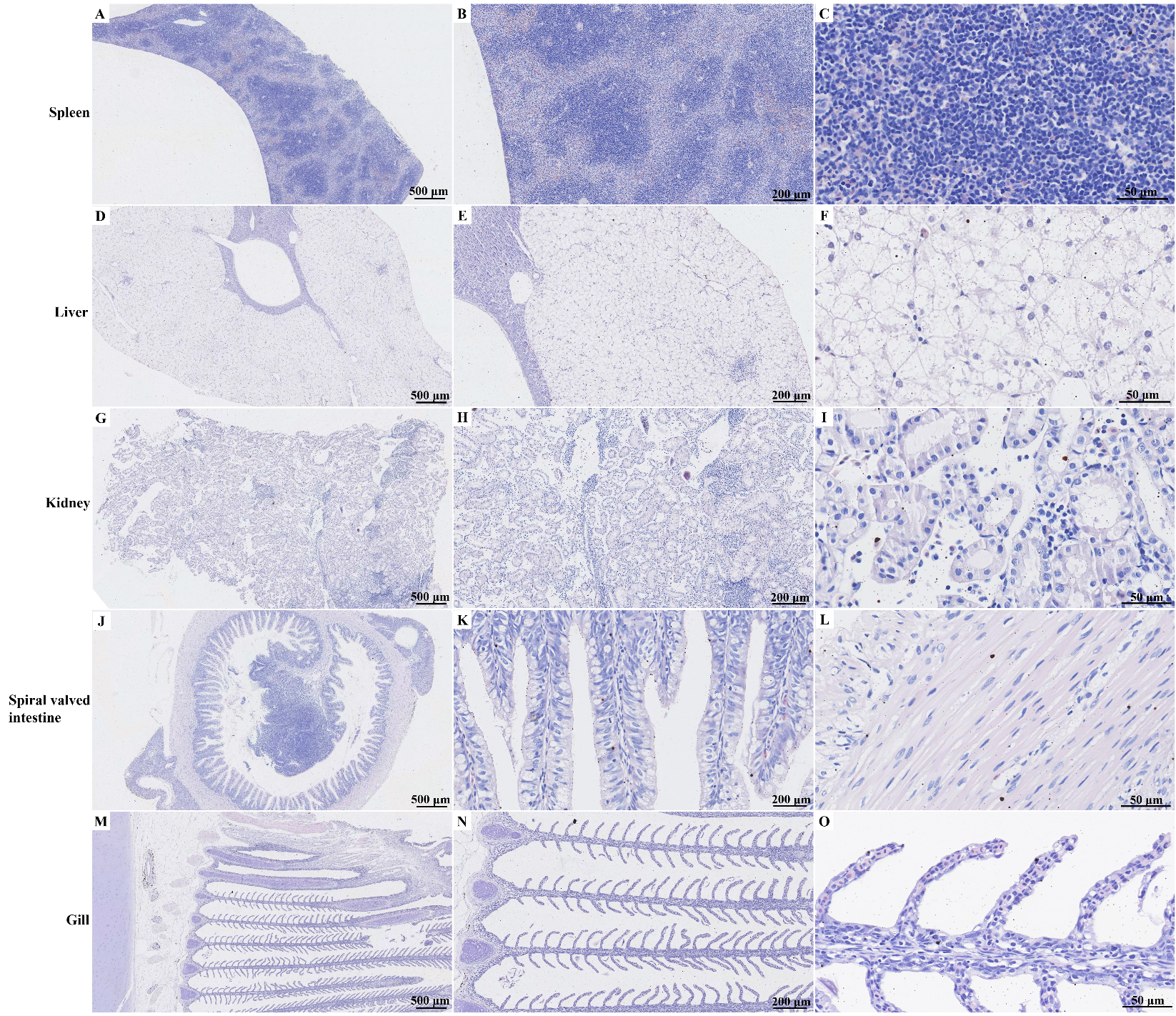
**Figure S2** Acid-fast staining of different tissues of control hybrid sturgeon. (A-C) Spleen; (D-F) Liver; (G-I) Kidney; (J-L) Spiral valved intestine; (M-O) Gill.

**
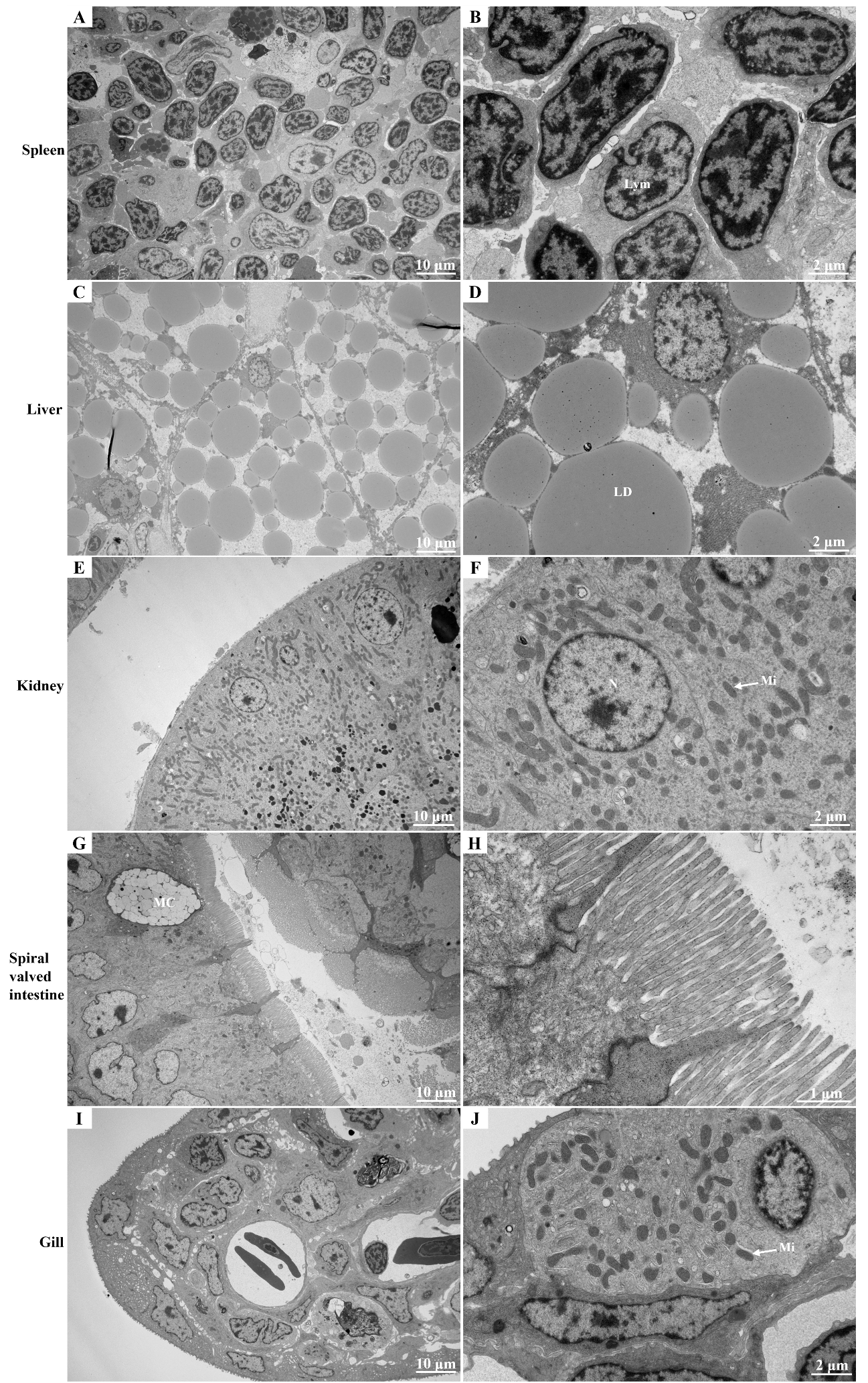
****Figure S3** Ultrastructure of different tissues of control hybrid sturgeon. (A-B) Spleen; (C-D) Liver; (E-F) Kidney; (G-H) Spiral valved intestine; (I-J) Gill. LD. lipid droplet; Lym. lymphocyte; MC. mucous cell; Mi. mitochondria.
